# The progression of inorganic nanoparticles and natural products for inflammatory bowel disease

**DOI:** 10.1186/s12951-023-02246-x

**Published:** 2024-01-03

**Authors:** Qingrong Li, Liting Lin, Cong Zhang, Hengguo Zhang, Yan Ma, Haisheng Qian, Xu-Lin Chen, Xianwen Wang

**Affiliations:** 1 Department of Pharmacology, School of Basic Medical Sciences, Anhui Medical University, Hefei, 230032 People’s Republic of China; 2 School of Biomedical Engineering, Research and Engineering Center of Biomedical Materials, Anhui Provincial Institute of Translational Medicine, Anhui Medical University, Hefei, 230032 People’s Republic of China; 3 Division of Gastroenterology, Division of Life Science and Medicine, The First Affiliated Hospital of USTC, University of Science and Technology of China, Hefei, Anhui 230026 People’s Republic of China; 4 Key Laboratory of Oral Diseases Research of Anhui Province, College and Hospital of Stomatology, Anhui Medical University, Hefei, 230032 People’s Republic of China; 5 Department of Burns, The First Affiliated Hospital of Anhui Medical University, Hefei, 230022 People’s Republic of China

**Keywords:** Inflammatory bowel disease, Inorganic nanoparticles, Natural product nanomaterials, ROS/RNS, M1 macrophages

## Abstract

There is a growing body of evidence indicating a close association between inflammatory bowel disease (IBD) and disrupted intestinal homeostasis. Excessive production of reactive oxygen species (ROS) and reactive nitrogen species (RNS), along with an increase in M1 proinflammatory macrophage infiltration during the activation of intestinal inflammation, plays a pivotal role in disrupting intestinal homeostasis in IBD. The overabundance of ROS/RNS can cause intestinal tissue damage and the disruption of crucial gut proteins, which ultimately compromises the integrity of the intestinal barrier. The proliferation of M1 macrophages contributes to an exaggerated immune response, further compromising the intestinal immune barrier. Currently, intestinal nanomaterials have gained widespread attention in the context of IBD due to their notable characteristics, including the ability to specifically target regions of interest, clear excess ROS/RNS, and mimic biological enzymes. In this review, we initially elucidated the gut microenvironment in IBD. Subsequently, we delineate therapeutic strategies involving two distinct types of nanomedicine, namely inorganic nanoparticles and natural product nanomaterials. Finally, we present a comprehensive overview of the promising prospects associated with the application of nanomedicine in future clinical settings for the treatment of IBD (graphic abstract).

**Graphical Abstract:**

**Figure:**
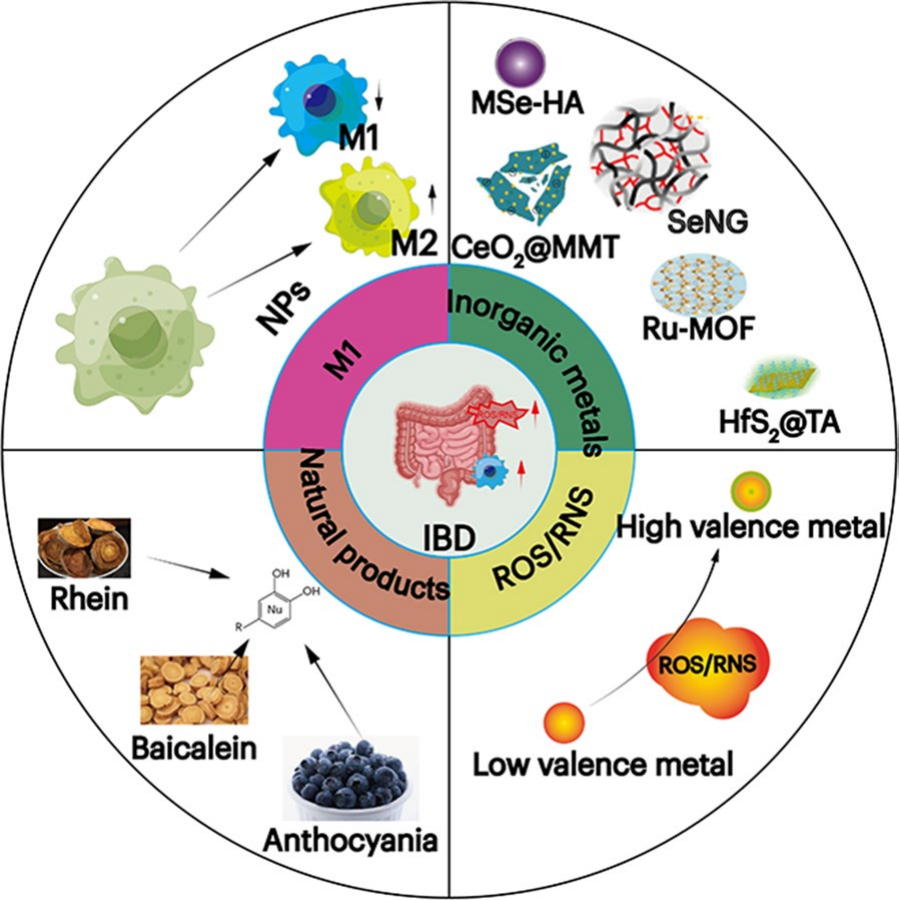
Different classes of nanomedicine are used to treat IBD. This review primarily elucidates the current etiology of inflammatory bowel disease and explores two prominent nanomaterial-based therapeutic approaches. First, it aims to eliminate excessive reactive oxygen species and reactive nitrogen species. Second, they focus on modulating the polarization of inflammatory macrophages and reducing the proportion of pro-inflammatory macrophages. Additionally, this article delves into the treatment of inflammatory bowel disease using inorganic metal nanomaterials and natural product nanomaterials

## Introduction

**Fig. 1:**
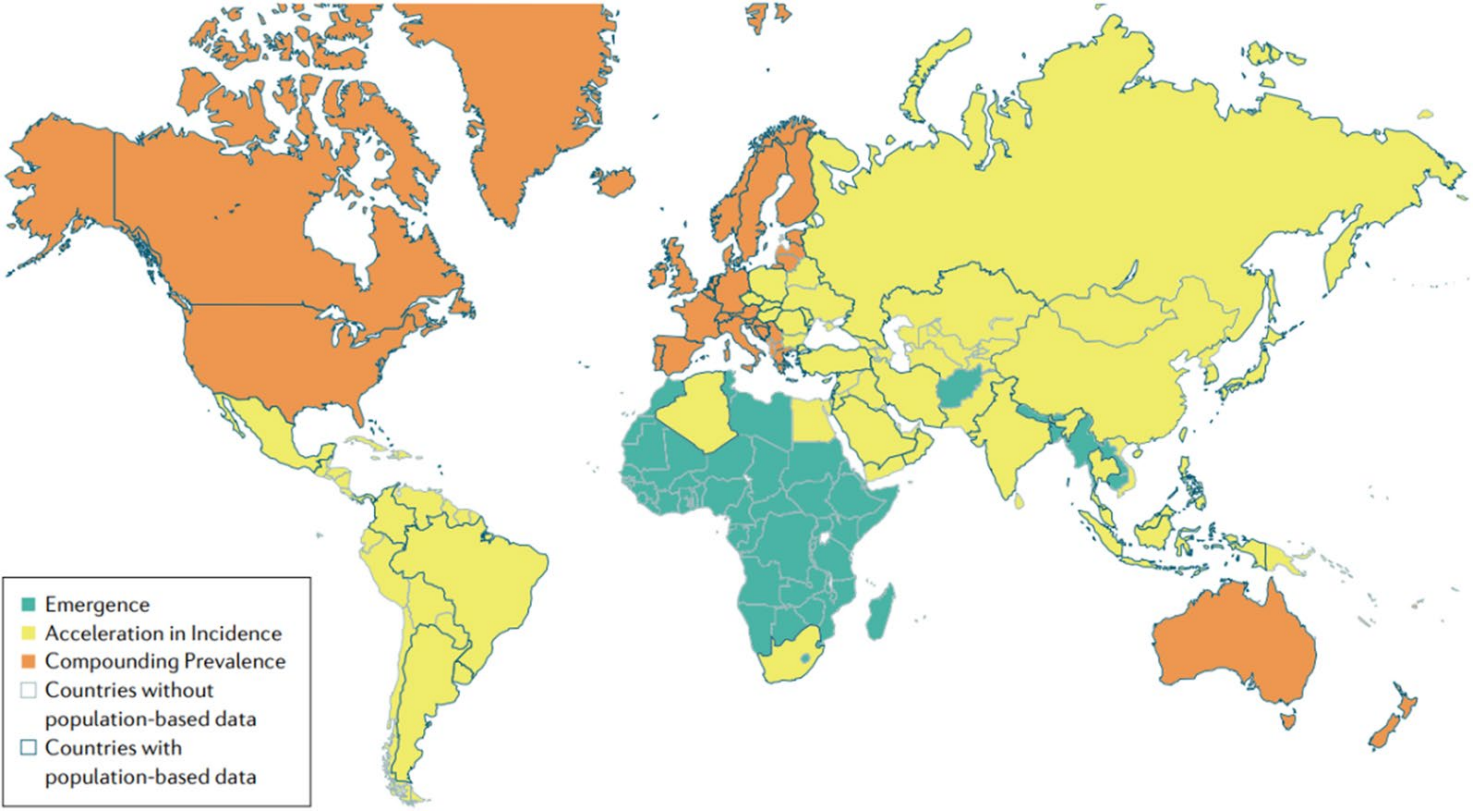
Global epidemiological stage map of IBD evolution in 2020. According to the United Nations Development Classification (2020), regions are organized according to the expected current epidemiological stage of the evolution of inflammatory bowel disease (IBD). Areas of the first stage (sudden onset) are highlighted in green, areas of the second stage (accelerated incidence) are highlighted in yellow, and areas of the third stage (complex epidemic) are highlighted in orange. Areas with black borders are areas where population-based incidence or prevalence studies can be conducted. Data on incidence or prevalence are lacking in areas with gary boundaries (cited in Kaplan et al. [4])

Inflammatory bowel disease (IBD), which includes ulcerative colitis and Crohn’s disease, is a chronic intestinal disorder characterized by symptoms such as diarrhoea, abdominal pain, anaemia, and weight loss. Over the past decade, the unprecedented rise in IBD cases has imposed a substantial financial burden on global public healthcare systems. The aetiology of this chronic disease remains elusive, although recent studies have elucidated a subset of IBD characterized by changes in the immune response and the microbiome. Genetic mutations, host immune dysfunction, mucosal barrier defects, and intestinal microbiota disorders are among the factors that can contribute to the development of this condition. Moreover, there is a higher prevalence of IBD in developed countries than in developing countries. Additionally, dietary changes have substantially contributed to the increased annual incidence of IBD observed in China [1–3]. According to a summary by “Nature Reviews Gastroenterology & Hepatology,” IBD is a global disease that evolves through four epidemiological stages: emergence, accelerating incidence, compound epidemic, and endemic equilibrium (Fig. 1). Developing countries are in the emergence stage with fewer recorded IBD cases. Emerging industrialized nations are in the accelerating incidence stage, which is characterized by a rising incidence rate but relatively low prevalence rate. Western countries are in the compound epidemic stage, as the incidence rate has stabilized but the prevalence rate is continually increasing [4]. Moreover, patients with colitis face an increased risk of developing colorectal cancer [5]. The rapid rise in the prevalence of IBD poses an increasingly serious threat to human health, making the treatment of IBD a pivotal medical research concern. Current clinical treatment modalities include aminosalicylate administration, steroid therapy, immunosuppressive agent administration, faecal microbiota transplantation, monoclonal antibody therapy, and surgical interventions. However, IBD patients still have an unfavourable prognosis, and there are widespread concerns about the side effects of these treatments. Consequently, a thorough exploration of IBD treatment and the development of new therapeutic strategies are needed to combat the pivotal challenges in current IBD management [6].

**Fig. 2:**
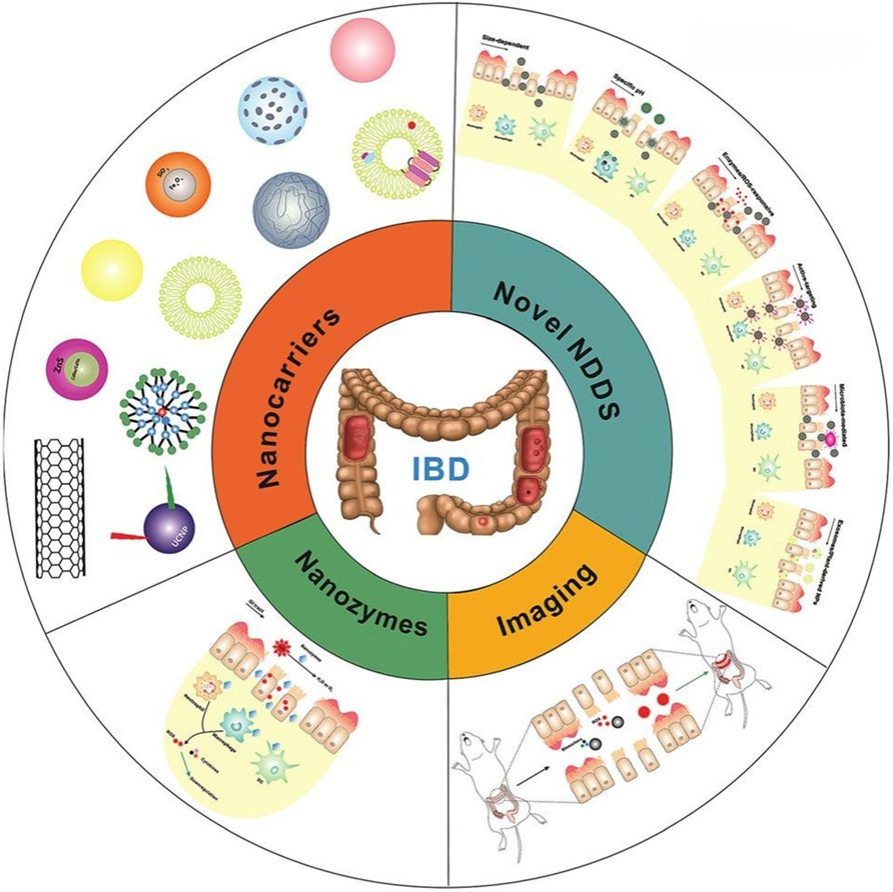
Application of nanoparticles in IBD treatment. In this paper, the types of nanocarriers, various new NDDSs, therapeutic effects of nanomases and progress in multimodal imaging of IBD are reviewed. *IBD* inflammatory bowel disease, *NDDSs* NP-based drug delivery system (cited in Yang et al. [10])

The integration of nanotechnology and biomedicine represents a breakthrough in disease treatment. Nanotechnology is used to combine small molecule drugs with organic or inorganic materials through physical or chemical interactions, creating drugs with nanosized dimensions. Compared to traditional drugs, nanodrugs possess several advantages due to their small size and large surface area [7, 8]. Nanodrugs can effectively enhance drug solubility and stability, improve drug targeting, facilitate drug absorption, and increase bioavailability. Drug delivery to inflamed intestinal areas is challenging because of not only the route of administration but also inherent pathological processes. For patients and clinicians, oral administration is the preferred route. However, oral drug delivery is also the most challenging due to various barriers and environmental factors affecting drug delivery to the target site. These barriers include pH fluctuations, the presence of multiple enzymes, the presence of food and microbiota in the digestive tract, intestinal motility, and mucus barriers [9]. In theory, colonic targeting strategies can enhance drug efficacy to some extent while reducing systemic side effects. Traditional colon-targeting approaches include extraintestinal, rectal, and oral administration. Although extraintestinal and rectal administration have shown favourable therapeutic effects, inevitable systemic absorption and challenges in precisely reaching the diseased area still occur. Moreover, traditional colonic targeting formulations are relatively large and exhibit poor penetration through mucosal barriers and cellular uptake [10]. The emergence of nanotechnology presents immense potential for overcoming these biological barriers. First, NPs can deliver drugs in a sustained and controlled manner and can be engineered to degrade and release their cargo in response to specific environmental stimuli. Second, surface modification of NPs can enhance their ability to target the intestinal microenvironment effectively. Third, the small size of nanocarriers allows more efficient targeting of diseased tissues by accumulating in inflamed and damaged epithelial cells through the enhanced permeation and retention (EPR) effect [11]. Due to the observed increase in vascular permeability and epithelial permeability in IBD, many studies suggest that reducing the size of drug delivery carriers to the nanoscale can improve the retention time of NPs in the inflamed intestinal region [12, 13]. To date, various nanodrugs have been investigated for the treatment of IBD, offering new directions and strategies for managing this disease. Figure 2 lists the types of nanodrugs that have been widely applied in the treatment of IBD [10].

Therefore, we will examine nanomedicine to explore alternative perspectives for the management of IBD. In this review, an overview of the mechanisms underlying intestinal inflammation is provided, and potential therapeutic strategies (such as modulation of M1 macrophages and elimination of ROS/RNS) are presented within the context of the IBD microenvironment. Subsequently, a comprehensive analysis is presented on the therapeutic mechanisms involving inorganic nanomaterials and natural product nanomaterials. It is anticipated that this study will undoubtedly guide future directions in utilizing nanomedicine for IBD management.

### IBD microenvironment: rich in reactive oxygen species (ROS), active nitrogen species (RNS), and proinflammatory M1 macrophages

The pathogenesis of IBD is multifaceted and involves the immune response, environmental factors, and genetic susceptibility. Extensive research has elucidated the critical functions and regulatory mechanisms of immune cells in the physiological and pathological processes of IBD. Different types of immune cells, such as macrophages, T cells, B cells, dendritic cells (DCs), and mesenchymal stem cells (MSCs), are present in the intestine and play crucial roles in early defence against intestinal pathogens. Macrophages act as the first line of defence against foreign antigens by recognizing pathogens, engulfing microbes, and regulating inflammation in the intestine to maintain their balance. In cases of IBD progression where the integrity of the intestinal mucosal barrier is compromised, macrophages can facilitate healing of the mucosal barrier and coordinate immune response development [14–16]. Macrophage polarization plays a role in the regulation of intestinal homeostasis and disease progression. Macrophages can be classified into two phenotypes based on their activation status: M1 macrophages, which exhibit proinflammatory characteristics, and M2 macrophages, which display anti-inflammatory properties [17, 18]. In IBD, there are notable distinctions in the phenotype and distribution of macrophages infiltrating the intestinal mucosa compared to tissue-resident macrophages during homeostasis [19]. M1 macrophages play a vital role in the engulfment of pathogens and microorganisms, which is essential for the resolution of inflammation and facilitation of wound healing [20]. M1 macrophages are responsible for the production of numerous cytokines and chemokines that contribute to the escalation of the inflammatory response and tissue damage. In contrast, M2 macrophages release anti-inflammatory cytokines, engage in postinjury tissue remodelling and repair, eliminate debris, and exhibit a greater capacity to facilitate vascular regeneration [21, 22]. Currently, the deployment of biological agents such as infliximab, adalimumab, ustekinumab, and vedolizumab is prevalent. Nonetheless, the effectiveness of these agents in certain patients with inflammatory bowel disease (IBD) may be limited by with notable potential side effects, such as infusion reactions, cardiovascular complications, pulmonary issues, dermatological manifestations, and hemorrhaging [23]. This variation in effectiveness is due to the necessity of M2 macrophages for a successful response to TNF inhibitors [24]. Consequently, there is immense potential in directing macrophage polarization and manipulating macrophase phenotype as a promising therapeutic approach for managing IBD.

**Fig. 3:**
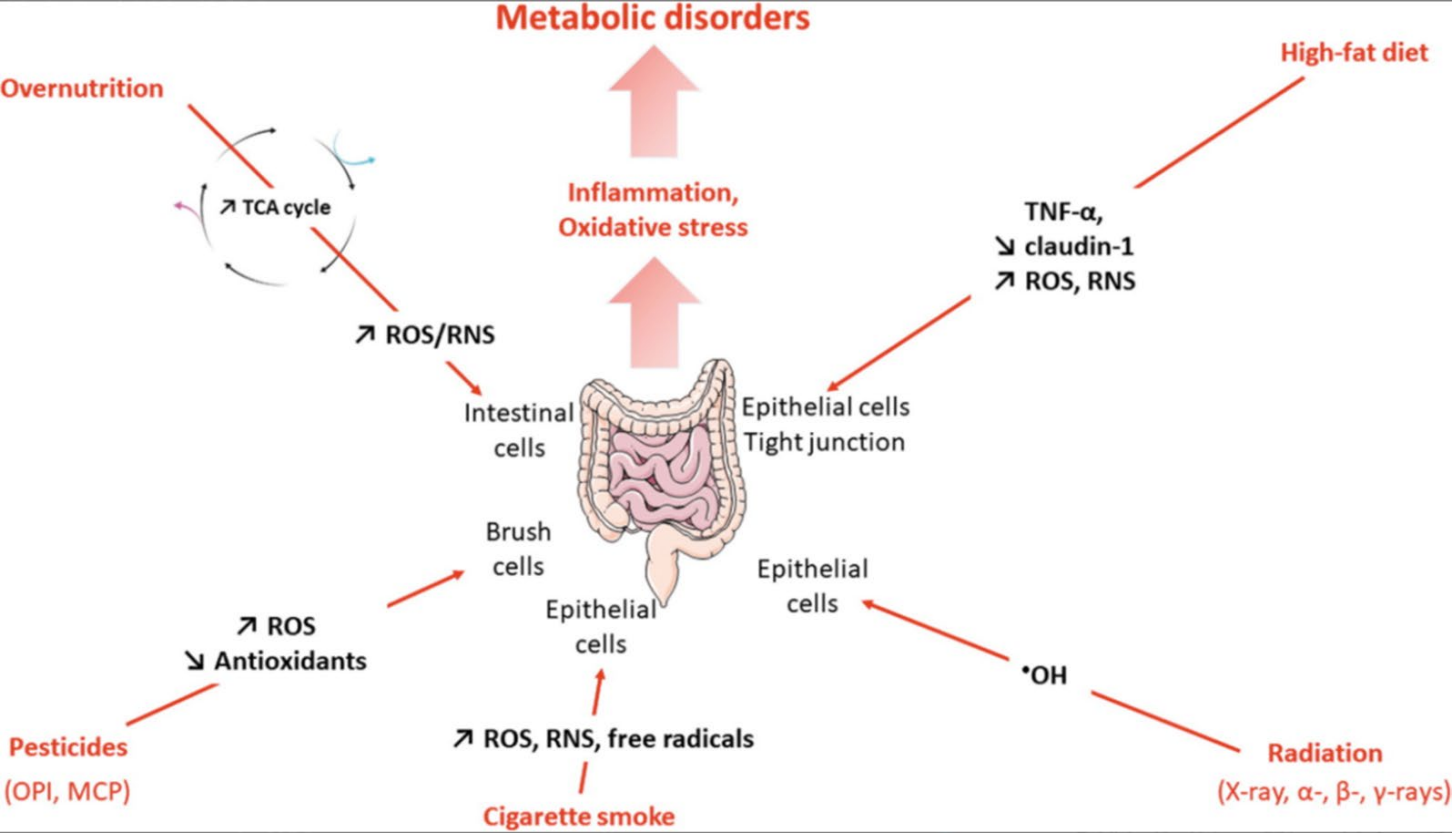
The origin of intestinal inflammation and oxidative stress in metabolic disorders. Overnutrition, high-fat diets, radiation, cigarette smoke, and pesticides all contribute to the production of ROS and RNS in the gut. These ROS and RNS further contribute to inflammation and oxidative stress in metabolic disorders. ·OH: hydroxyl radical; MCP: monocrotophos; OPI: organophosphorus insecticide; Tricarboxylic acid (TCA); TNF-α: tumor necrosis factor-α (cited in Abot et al. [39])

While the exact causes and development of IBD remain uncertain, it is known that there is notable production of ROS/RNS at the sites where colonic inflammation occurs. The excessive generation of these ROS/RNS, coupled with subsequent oxidative stress and redox regulation, has been shown to play a critical role in the pathophysiology of IBD in both experimental animal models and IBD patients (Fig. 3). ROS/RNS include hydroxyl radicals (·OH) and superoxide anions (O^2−**.**^), nitric oxide (NO), and peroxynitrite (OONO–), are pivotal in the progression of colonic inflammatory diseases [25, 26]. ROS not only act as signalling molecules but also serve as inflammatory mediators [27]. Numerous studies on animals and patients have demonstrated that increased ROS levels can lead to oxidative harm to proteins, lipids, and DNA. This damage can exacerbate the progression of intestinal inflammation, mucosal injury, and ulceration in IBD patients. Moreover, endogenous antioxidants are often insufficient to effectively eliminate overexpressed ROS. Therefore, the use of exogenous antioxidants to target excessive ROS and maintain oxidative balance within the body, commonly referred to as antioxidant therapy, has been considered one of the most promising treatment options for preventing and managing various oxidative stress-related diseases [28].

RNS include representative molecules such as NO, which plays a crucial role in the human body as a free radical gases. Researchers believe that NO regulates inflammation, including processes such as leukocyte migration and the transcription of proinflammatory genes [29]. In addition to damaging host tissues, NO has cytotoxic effects on not only microbes but also the cells producing it and neighbouring cells. NO regulates immunity by inducing host immune cell death. In addition to its cytotoxicity, NO can also form complexes with superoxide radicals, leading to oxidative damage in target cells and host cells [30]. Simultaneously, NO is a natural anti-inflammatory mediator secreted by macrophages and, under normal physiological conditions, kills bacteria and viruses through the disruption of metabolic enzymes and DNA [31, 32]. However, NO, as an inflammatory mediator, can cause tissue damage and cell apoptosis in physiological disorders [33]. Elevated levels of NO over an extended period are associated with severe chronic inflammatory diseases such as IBD, sepsis, rheumatoid arthritis, and systemic lupus erythematosus [34–37]. The biological activity of NO can mediate the pathological effects of cytokines such as IL-6 and TNF-α, resulting in tissue damage and inflammation. Excessive NO production can lead to various consequences, including damage to the intestinal mucosa and epithelial barrier, inhibition of intestinal motility, and intestinal inflammation (Fig. 3) [38, 39]. Therefore, the removal of ROS/RNS is considered an effective strategy for the treatment of colitis [40, 41].

## Nanomedicine for the treatment of IBD

### Inorganic metals

Researchers have made substantial progress in the application of inorganic NPs for the treatment of IBD by improving the synthesis and modification of these NPs. Inorganic NPs exhibit remarkable catalytic properties and enzyme-like activities, allowing them to directly react with ROS and RNS and exert potent therapeutic effects against oxidative stress damage. The catalytic properties of inorganic NPs are considerably influenced by various physical and chemical parameters, such as their size, surface charge, surface area, and surface ligands. The excretion and distribution of NPs in the body are closely related to their size. When epithelial barriers are disrupted, positively charged proteins are exposed, attracting negatively charged inorganic NPs, which promotes NP accumulation at inflammatory sites. Inorganic NPs with a larger surface area provide more active sites for catalysis. Modifying the ligands can improve the stability, water solubility, and biocompatibility of inorganic NPs. Nanoantioxidants have shown great potential for clinical applications and have been extensively studied. However, there are still challenges to overcome in terms of synthesizing and purifying these compounds through multiple steps due to their potential toxicity and high cost. In the following discussion, we will discuss in detail the development of inorganic NPs for IBD treatment [11–13].

#### Selenium NPs

Selenium is an essential trace element for mammals and plays a crucial role in maintaining human health. An increasing body of evidence suggests a close association between selenium deficiency and various physiological disorders, including chronic inflammatory diseases, heart dysfunction, vascular blockage, and cancer. Recent research has indicated a significant negative correlation between selenium levels and the severity of ulcerative colitis. Selenium has been proposed as a noninvasive biomarker for IBD diagnosis and severity [42, 43]. The multifunctional effects of selenium in the human body are primarily achieved by incorporating selenocysteine (SeC) into selenoproteins, a protein family that has 25 members. Selenoproteins play vital roles in antioxidant defences, as well as in immune and inflammatory regulation [44–47].

**Fig. 4:**
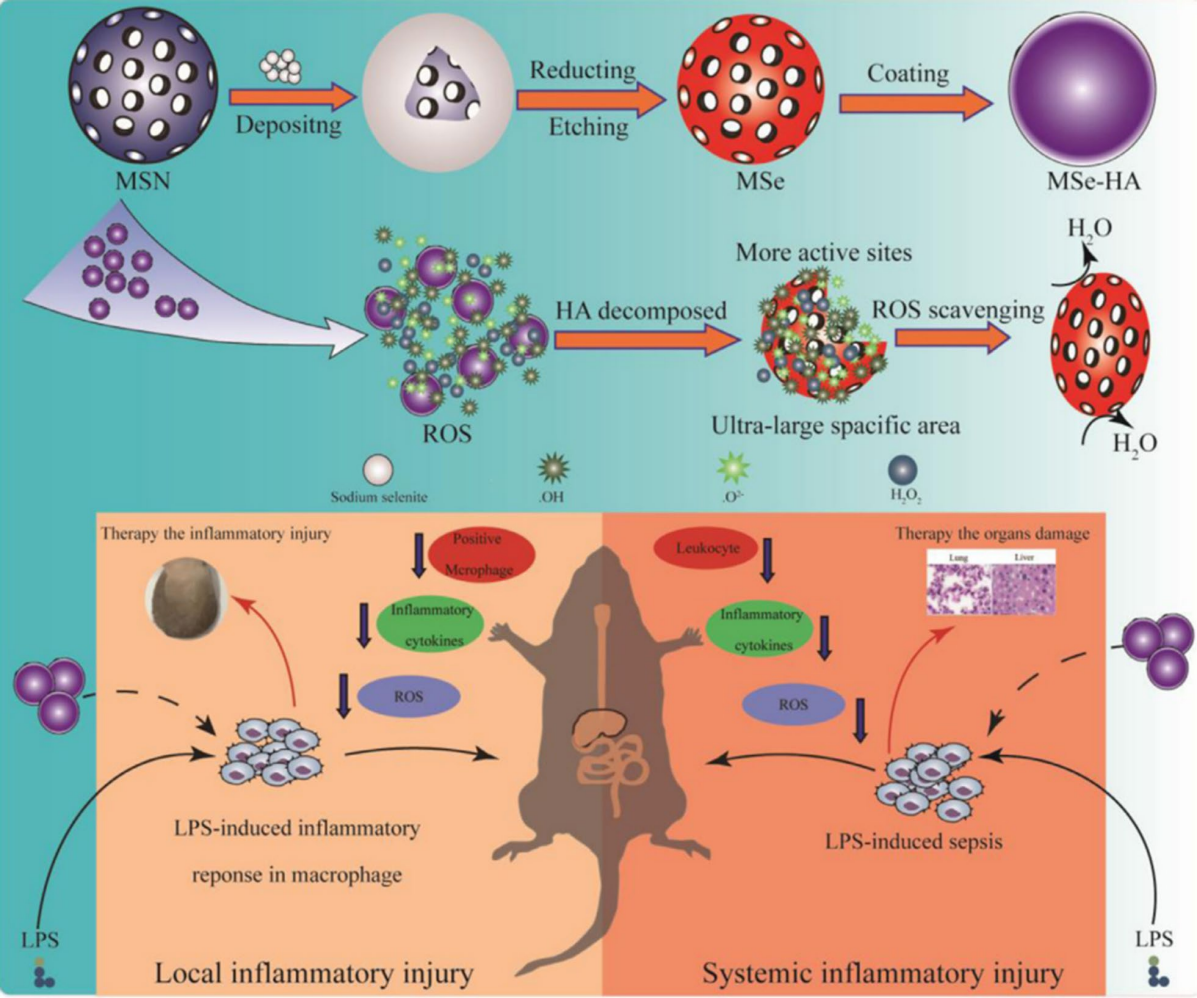
Schematic diagram of the protective effect of MSe-HA NPs in a colitis model. Construction of MSe-HA NPs and the use of MSe-HA NPs in the treatment of local and systemic inflammatory injuries (cited in Chen et al. [47])

Organic selenium compounds, such as glutathione peroxidase (GPx), are natural molecules widely used in organic synthesis. Selenium compounds with GPx enzyme activity catalyse the reduction of peroxides and maintain the metabolic balance of ROS [48, 49]. The use of nanomaterials synthesized from organic selenium compounds for the treatment of inflammatory diseases has been extensively tested. For instance, mesoporous silica NPs (MSNs), characterized by their high surface area, have been used as a delivery system for the transport of selenium compounds with GPx enzyme activity. Furthermore, this nanoparticle system has been modified with hyaluronic acid (HA) to precisely target inflammatory macrophages. Similarly, high-surface-area mesoporous selenium NPs exhibit robust hydrogen peroxide (H_2_O_2_) removal abilities, with an efficiency 2.02 times greater than that of solid selenium nanoparticles. These compounds have demonstrated promising therapeutic effects in mouse models of colitis and sepsis (Fig. 4) [50]. Additionally, biogenic selenium NPs (BNS) synthesized using *Enterobacter cloacae* Z0206 have been employed. These BNS activate the Nrf2 antioxidant pathway, protecting the intestinal barrier from oxidative stress-induced damage.

**Fig. 5:**
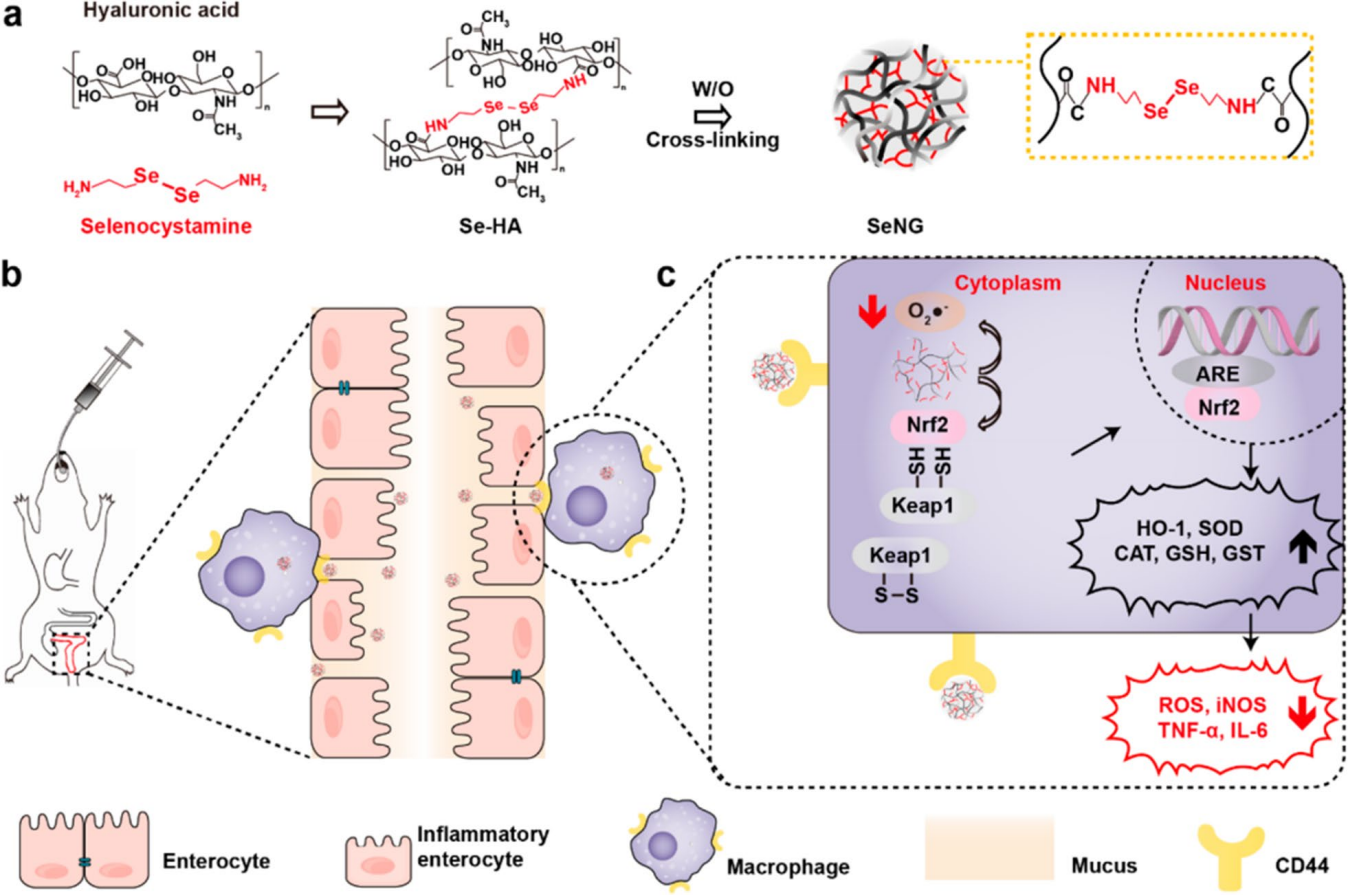
Schematic diagram of the protective effect of SeNG in a colitis model. **a** A simple process for the synthesis of SeNG by emulsion. **b** The high binding affinity between HA and CD44 at the disease site enables effective inflammatory targeting. **c** In terms of mechanism, SeNG effectively clears ROS by directly eliminating and regulating the Nrf2 signaling pathway in the nucleus SeNG significantly up-regulates the expression of anti-ROS proteins (such as HO-1, SOD, CAT, GSH, and GST), and down-regulates the expression of iNOS and inflammatory factors (such as TNF-α and IL-6) (cited in Xu et al. [53])

Selenium-based compounds are widely recognized for their antioxidant properties and are crucial for regulating cellular redox processes. Se can easily undergo oxidation to form seleninic acid or be reduced back to its original diselenide state depending on the prevailing redox conditions [51, 52]. Surprisingly, in a state of normal bodily functions, selenates have the ability to utilize oxygen and subsequently transform into diselenide via a process of dehydration [53, 54]. In this particular scenario, Professor Nie Guangjun and his research team put forward a hypothesis that the specially designed diene amide-bridged hyaluronic acid nanogel (SeNG) could potentially offer a solution to combat the excessive build-up of ROS at sites of tissue inflammation in individuals with IBD. The addition of hyaluronic acid (HA) enhances the nanoplatform’s targeting capabilities for CD44-overexpressing inflammatory cells, particularly macrophages. When administered orally to a colitis mouse model, the nano-antioxidant SeNG accumulates at the inflammatory site, especially in CD44+ immune cell-rich areas. The nanogel effectively protected the colonic epithelium and reduced inflammation by clearing reactive oxygen species (ROS). Additionally, SeNG enhances the Nrf2/HO-1 signaling pathway, leading to increased levels of antioxidant enzymes such as superoxide dismutase (SOD), catalase (CAT), glutathione (GSH), and glutathione *S*-transferase (GST) (Fig. 5) [55].

The research team led by Professor Huang Zhi prepared a highly potent anti-inflammatory component, mannose-rich oligosaccharides (MRO), through enzymatic hydrolysis. MRO was then loaded into functionalizing selenium NPs (SeNPs) to treat inflammation in an ulcerative colitis animal model by accelerating macrophage reprogramming. In vitro, MRO-SeNPs reprogrammed macrophages, which exhibited a typical M2 polarization cytokine expression profile. Furthermore, in a dextran sulphate sodium (DSS) mouse model, MRO-SeNPs effectively alleviated colitis by locally suppressing inflammatory cytokines such as IL-1β, IL-6, TNF-α, IL-12, and MCP-1. They also demonstrated robust antioxidant capabilities within colonic tissues [56].

#### Cerium dioxide NPs

**Fig. 6:**
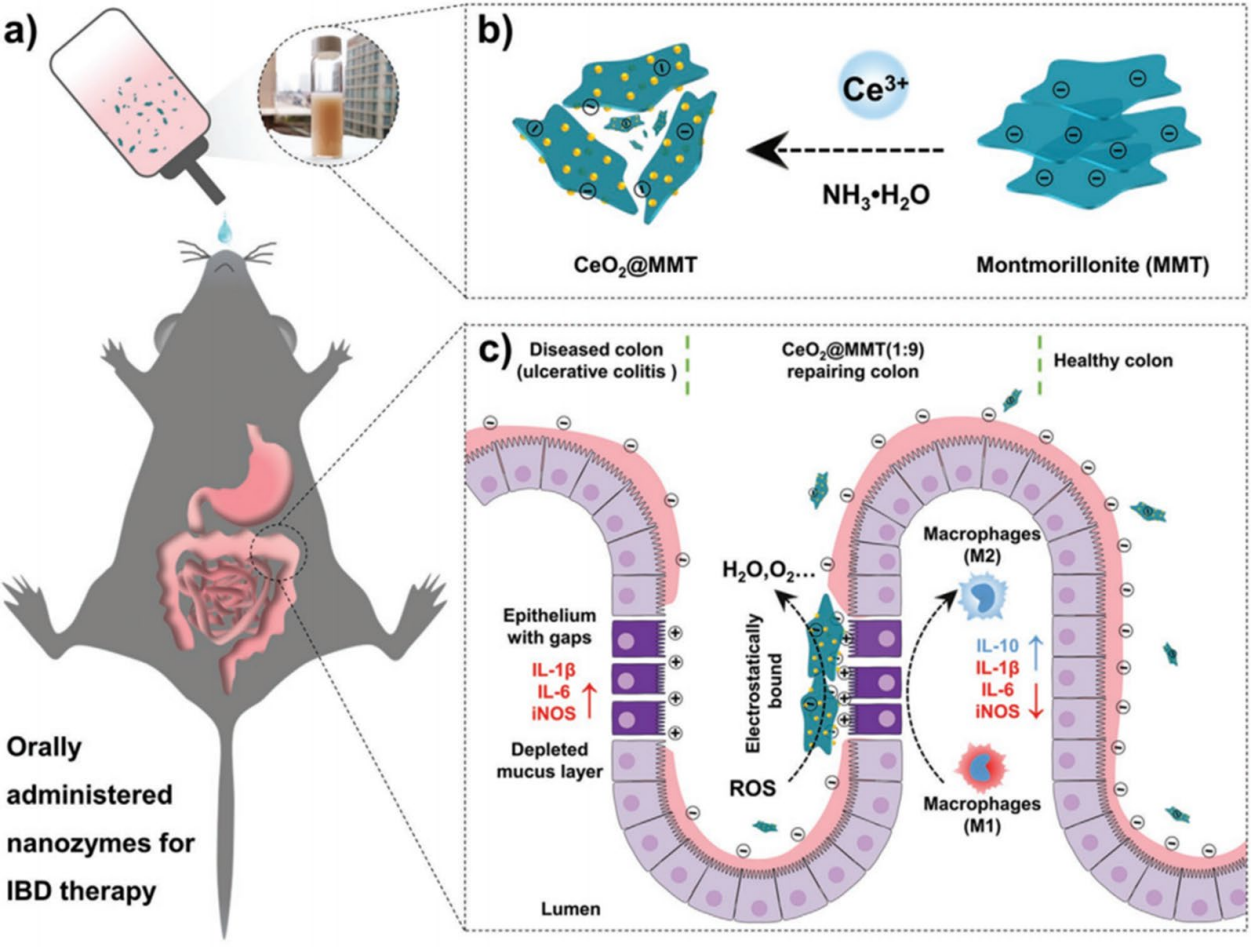
Design and synthesis of CeO_2_@MMT for the oral treatment of inflammatory bowel disease (IBD). **a** CeO_2_@MMT oral treatment of IBD mice. **b** A scheme for synthesizing CeO_2_@MMT by in-situ growth of cerium dioxide nanoparticles (CeO_2_ NPs) onto MMT sheets. **c** The therapeutic effect of CeO_2_@MMT on IBD. Left: Ulcerative colitis is characterized by mucous depletion, positively charged protein accumulation, increased permeability of the epithelial cell layer, elevated ROS and pro-inflammatory macrophage (M1) levels, and upregulation of cytokines (IL-1β, IL-6, iNOS, etc.). Middle: the negatively charged CeO_2_@MMT (1:9) specifically targets the positively charged inflammatory colon. When electrostatic binding, CeO_2_@MMT removes elevated ROS through the multiple enzymatic functions of CeO_2_ NPs. Right: CeO_2_@MMT (1:9) After intervention, the damaged intestinal epithelial barrier was partially repaired, pro-inflammatory macrophages (M1) and cytokines (IL-1β, IL-6, iNOS, etc.) were reduced, and anti-inflammatory macrophages (M2) and cytokines (such as IL-10) were increased (cited in Zhao et al. [62])

Cerium dioxide (CeO_2_) NPs have received attention due to their antioxidant activity endowed by the variable oxidation states of cerium (Ce^3+^/Ce^4+^). They have been reported to act as ROS/RNS scavengers in the treatment of spinal cord injuries, inflammation, sepsis, Alzheimer’s disease, ischaemic stroke, and Parkinson’s disease [57–62]. Professor Feng Zeng’s research group synthesized hydrophobic ultrafine cerium NPs (CeNPs) and modified them with PEG to create CeNPs-PEG. CeNPs-PEG effectively treat DSS-induced colitis by decreasing proinflammatory macrophage numbers and suppressing Th1/Th17 responses, thereby improving the proinflammatory microenvironment. It achieved this by inhibiting the coactivation of the NF-κB and JAK2/STAT3 pathways [63]. In another study, Professor Wei Hui’s team aimed to target the site of colonic lesions by binding cerium oxide nanoenzymes with specific targeting agents, enhancing their efficacy against inflammatory bowel disease. They optimized CeO_2_@MMT (1:9) by assembling cerium oxide with montmorillonite (MMT), which can stably exist in the stomach and target the inflamed colon through electrostatic interactions. These NPs efficiently reduced inflammation by clearing ROS as a result of the MMT modification. The MMT modification increased the ability of CeO_2_ NPs to target the inflamed intestinal region, as indicated by transmission electron microscopy images of healthy mouse mucosa. This research demonstrated the excellent anti-inflammatory effects and precise targeting of CeO_2_@MMT (Fig. 6) [64].

Professor Wang Lin’s research group synthesized NPs with superoxide dismutase (SOD) and catalase (CAT) enzyme-like activities (Au/CeO_2_) for the treatment of IBD. These Au/CeO_2_ NPs possessed a core–shell structure and porous design, which allowed for a larger surface area to expose catalytic sites. This larger surface area promoted the redox reactions of cerium (III)/cerium (IV) and enhanced the antioxidant enzyme activity of cerium. By coating these NPs with negatively charged HA, known as Au/CeO_2_@HA, the NPs were shown to accumulate in inflamed colonic tissues after oral administration. Consequently, these compounds effectively reduced the production of proinflammatory cytokines and alleviated colonic damage in mice with acute colitis. These findings indicated that Au/CeO_2_@HA shows promising potential as a nanoantioxidant for treating IBD [65].

**Fig. 7:**
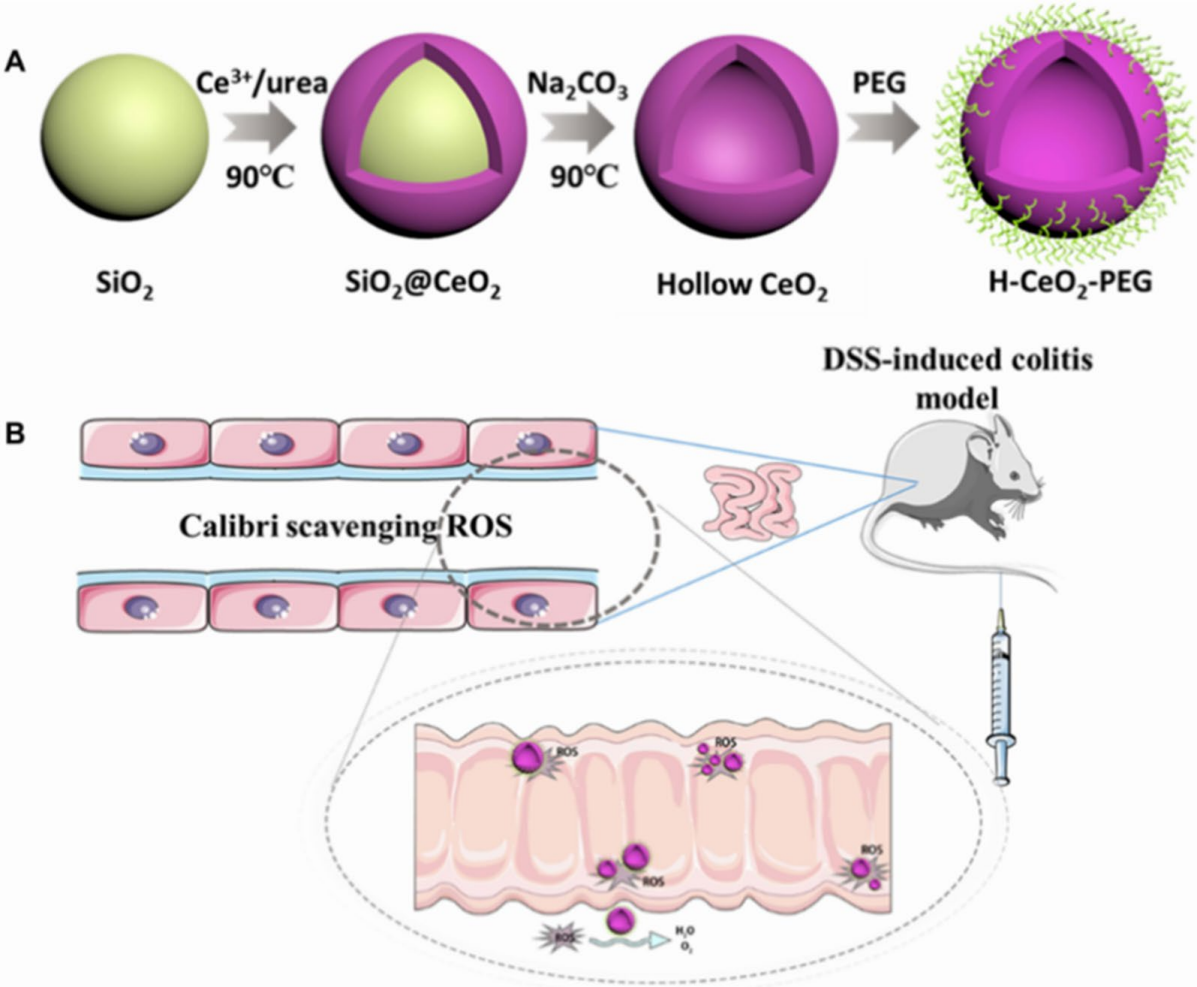
Diagram of H-CeO2-PEG in the treatment of colitis in mice. **A** Diagram of the preparation process of H-CeO2-PEG composites. **B** Schematic diagram of the effect of H-CeO2-PEG on DSS induced colitis in mice (cited in Yang et al. [65])

Professor Ma Guanghui’s research group employed extracellular vesicles as templates and carriers to enhance the therapeutic efficacy of nanoantioxidants. They developed a mild one-pot method that allowed cerium oxide NPs to grow in situ on the surface of red blood cell extracellular vesicles (Ce-ReVs). In a colitis model, Ce-ReVs significantly accumulated at inflammatory sites and exhibited potent ROS scavenging capabilities. Furthermore, by hybridizing ReVs with exosomes derived from mesenchymal stem cells, the system was upgraded, demonstrating additional reparative functions for highly damaged tissues [66]. Professor Ge BuJun’s research group synthesized uniform and monodisperse hollow CeO_2_ NPs (H-CeO_2_) using a hard template strategy and modified them with NH_2_-PEG-NH_2_ for colitis treatment. Their research findings revealed that H-CeO_2_-PEG can serve as an artificial nanoenzyme to clear ROS, alleviate DSS-induced colitis in mice, and suppress the production of inflammatory cytokines. H-CeO_2_-PEG also alleviated inflammation through the ERK1/2/JNK/P38/c-Jun signalling pathway (Fig. 7) [67]. Professor David P. Cormode’s research team has published findings indicating that dextran-coated cerium oxide NPs (Dex-CeNPs) exhibit potential as computed tomography (CT) imaging agents for IBD and possess cell-protective properties against oxidative damage. The Dex-CeNPs were synthesized through precipitation techniques and subjected to comprehensive characterization using diverse analytical methods. In vivo CT imaging experiments were also conducted on both healthy mice and those with DSS-induced colitis, demonstrating the ability of Dex-CeNPs to generate robust CT signals while selectively accumulating in the affected area. Moreover, within a 24-h timeframe, approximately 97% of the administered oral dosage was effectively eliminated from the body. Consequently, Dex-CeNPs hold promise as effective CT imaging agents for visualizing gastrointestinal complications associated with IBD while concurrently mitigating oxidative harm [68].

#### Tungsten NPs

**Fig. 8:**
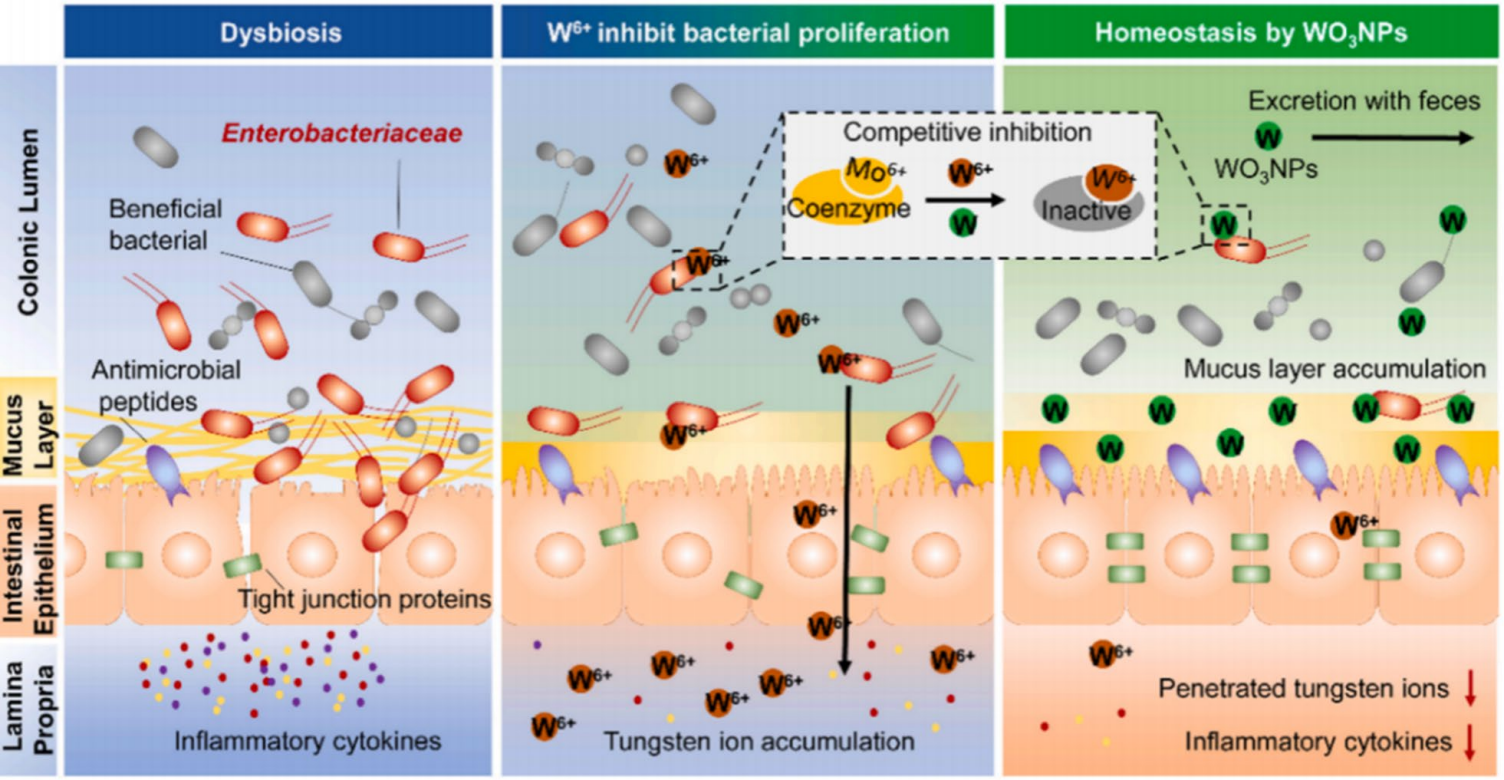
WO_3_ NPs Model and mechanism of mediated mucous layer accumulation and microbial metabolic reprogramming in the treatment of DSS induced colitis in mice. Oral WO_3_ NPs mainly accumulated in the mucous layer of the colon, and significantly inhibited the growth of pathogenic enterobacteriaceae in the colon of DSS induced colitis mice. WO_3_ NPs treatment induces significant limitations in bacterial respiratory and metabolic processes by competitively inhibiting the activity of mostreterinase and eliminating the anaerobic nitrate reductase activity of Enterobacteriaceae, thereby reducing intestinal inflammation, attenuating bacterial translocation, restoring the colon epithelial barrier, and remodeling intestinal flora homeostasis in the inflaming colon (cited in Qin et al. [67])

Tungsten therapy has gained considerable attention as a strategy to inhibit molybdenum cofactor-dependent microbial respiration pathways while reprogramming the gut microbiota during intestinal inflammation. IBDs are often associated with dysbiosis, which is characterized by changes in the gut microbiota composition, including the expansion of *Enterobacteriaceae (Enterobacteriales)* facultative anaerobes [69–71]. Therefore, inorganic sodium tungstate NPs have been developed to target Enterobacteriaceae for the treatment of colitis. Sodium tungstate can selectively inhibit molybdenum cofactor-dependent microbial respiration pathways that are only active during inflammatory episodes [72, 73]. Therefore, the development of nanomaterials specifically targeting Enterobacteriaceae in the gut microbiota has become more prevalent. Professor Nie Guangjun’s research group prepared monoclinic WO_3_ NPs by using annealed hexagonal ammonium tungstate (NH_4_WO_3_) and sodium borohydride (NaBH_4_) (Fig. 8). These NPs significantly improved bacterial richness and community diversity in DSS-induced colitis model mice compared to those in sodium tungstate treated mice. Furthermore, the inhibitory effect on Enterobacteriaceae was significantly enhanced. This nanomaterial used against Enterobacteriaceae significantly improved microbiota erosion, reduced intestinal inflammation, and restored the intestinal mucosal barrier [69]. Similarly, Professor Shi Jinjin’s research group, inspired by the binding of *Escherichia coli* strains to yeast cell wall mannans (YCW), used YCW as a biomimetic membrane to encapsulate NPs for enhanced targeting and colitis alleviation [74]. YCW was selected as a shell to encapsulate CaWO_4_. The YCW shell endows CaWO_4_ with excellent resistance to the harsh gastrointestinal environment and allows it to adhere to abnormally expanded *Escherichia coli* in colitis, serving as a targeting agent. Consistent with the finding of previous reports, DSS-induced colitis resulted in decreased microbiota diversity and abundance. However, compared to those in the model group, the group treated with CaWO_4_@YCW had significantly improved gut microbiota in terms of abundance and diversity. CaWO_4_@YCW also showed great potential for successfully restoring healthy microbiota [75].

**Fig. 9:**
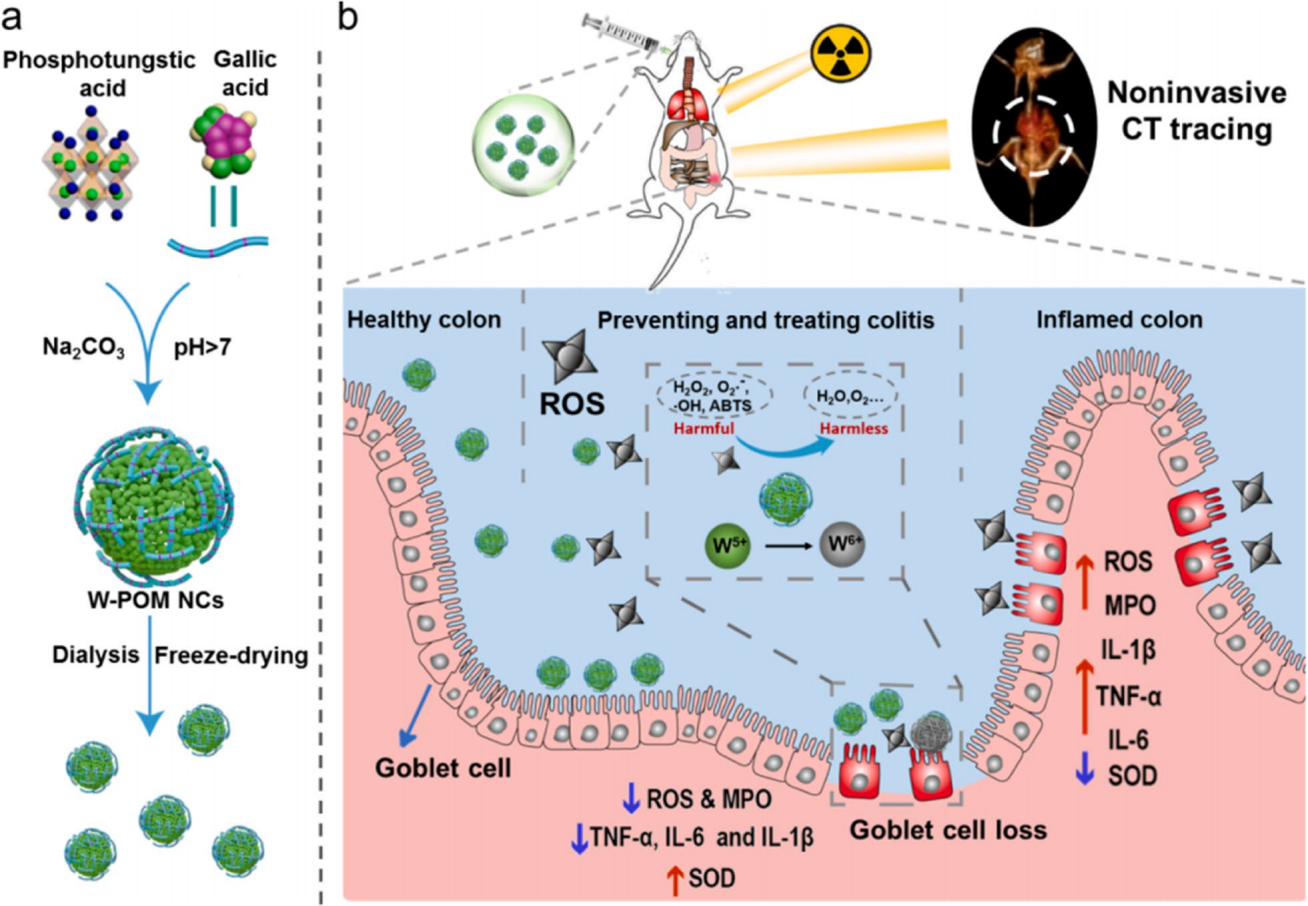
Schematic diagram of the protective effects of W-POM NCs in a colitis model. **a** Preparation of W-POM NCs and **b** schematic of the potential mechanisms of CT-traceable mitigation of colonic inflammation (cited in Wang et al. [76])

Professor Wang Hua’s research team has developed nanoclusters of polyoxometalate based on tungsten (W-POM NCs) for the prevention and treatment of IBD. The oral administration of W-POM NCs effectively treated acute colitis in a mouse model induced by DSS. Tungsten possesses a high capacity for X-ray absorption, enabling the biological distribution of W-POM NCs to be noninvasively tracked through CT imaging. W-POM NCs demonstrated remarkable stability in acidic gastric fluid and exhibited strong ROS scavenging capabilities, allowing them to reach the colon region successfully and alleviate inflammation. This treatment significantly reduced disease activity index (DAI) scores, decreased the expression levels of inflammatory cytokines, alleviated colonic damage, and mitigated weight loss (Fig. 9) [76].

#### Ruthenium (Ru) NPs

**Fig. 10:**
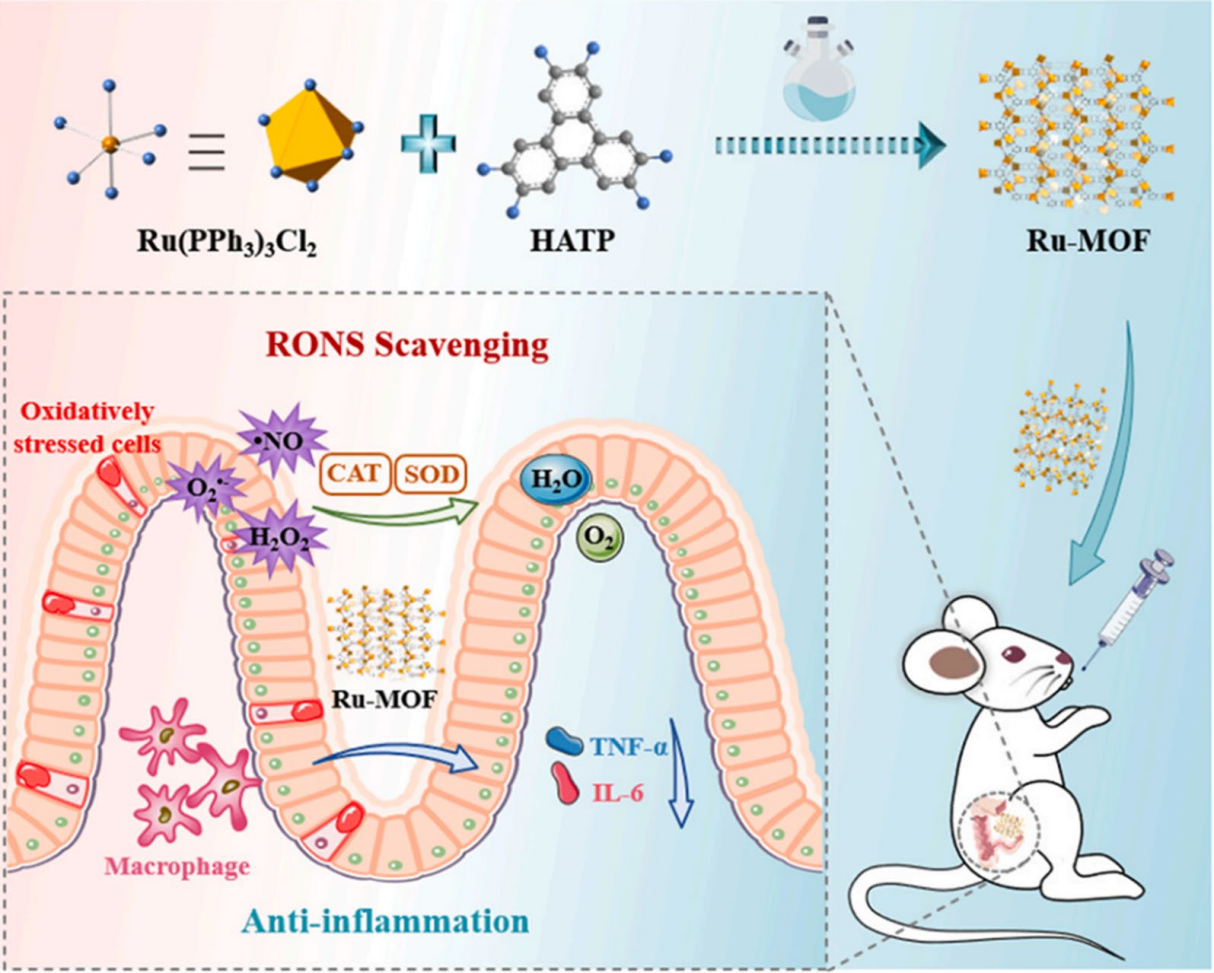
Schematic diagram of Ru-MOF in the treatment of RONS related diseases. *CAT* catalase, *SOD* superoxide dismutase, *TNF-α* tumor necrosis factor-α, *IL-6* interleukin-6 (cited in Liu et al. [78])

Ru is a member of the platinum group and belongs to the category of transition metals. It possesses redox activity, which allows for the conversion between Ru (II) and Ru (III), making it useful for ROS scavenging. Furthermore, studies have indicated that Ru complexes can function as scavengers for RNS such as NO and DPPH radicals. Wang Zheng’s research team successfully synthesized RU-based metal–organic frameworks (Ru-MOFs) using a straightforward hydrothermal method. They utilized a multidentate nitrogen-containing ligand called 2,3,6,7,10,11-hexaaminotriphenylene (abbreviated as HATP) along with Ru (PPh_3_)_3_Cl_2_ as the starting material. The resulting Ru-HATP MOF exhibited a layered structure with an average diameter of 141.6 nm. In vitro experiments demonstrated that Ru-MOF displayed exceptional capabilities in scavenging ROS/RNS while also providing protection against oxidative stress in cells. Additionally, it exhibited inhibitory effects on the expression of genes associated with inflammation. Notably, when tested in animal models with delayed treatment administration, significant therapeutic effects were observed (Fig. 10) [77, 78].

#### Zinc (Zn) NPs

Zn is a vital trace element within the human body, and its insufficiency is linked to immune disorders and neurological conditions such as Alzheimer’s disease. Research has indicated that Zn deficiency can result in intracellular oxidative stress, leading to cellular dysfunction. Moreover, Zn plays a critical role in maintaining intracellular redox homeostasis. IBD is characterized by an imbalance in free radicals, but studies have shown that supplementing with Zn can reduce intestinal permeability and reverse IBD-related risks [79]. Li Jinqua’s research group and others have demonstrated that ZnO NPs possess the ability to continuously release Zn^2+^, effectively inhibiting the progression of IBD. ZnO NPs, depending on the administered dose, can significantly reduce colon shortening as well as pathological damage. As antioxidants, ZnO NPs are capable of regulating factors associated with oxidative stress, such as ROS, the transcription factor Nrf2, and the antioxidant enzyme quinone oxidoreductase-1 (NQO-1) [80]. In addition, Riham Abdel-Hamid Haroun’s research team developed Zn NPs coated with frankincense acid (BAs-ZnNPs) to investigate their therapeutic effects on IBD. These findings indicated that BAs-ZnNPs exhibited anti-inflammatory properties in an experimental model of colitis. This anti-inflammatory effect was evident from the improvements observed in ALP, IgM, and IgG levels, as well as in the expression levels of NF-κB and COX-2 in both histological and biochemical experiments. The potential mechanisms involve the regulation of NF-κB and COX-2 gene expression, along with STAT-3 and PI3K protein expression, leading to a reduction in ALP, IgM, IgG and IL-8 levels in a rat model of ulcerative colitis [81].

#### Gold NPs

Gold-based materials have garnered considerable attention in the field of biomedicine due to their excellent biocompatibility, favourable renal clearance rates, and unique physicochemical properties [82–84]. Furthermore, as emerging nanomedicines, gold-based materials possess remarkable catalytic activity in clearing ROS and have been successfully applied in the treatment of IBD [85].

A research team led by Yin Junjie conducted a study on mice with IBD to assess the effectiveness of different coatings and sizes of AuNPs. The results showed that orally administered Au-5 nm/citrate and Au-5 nm/PVP had significant anti-colitis effects, as they were able to regulate colonic MPO enzyme activity, reduce IL-6 and TNF-α levels, act as catalysts for peroxynitrite and H_2_O_2_ decomposition, scavenge ROS/RNS, inhibit NF-κB activation, and decrease inflammatory factor levels. In vitro studies using RAW 264.7 cells also demonstrated that AuNPs could lower iNOS expression levels and NO production while mitigating LPS-induced NF-κB activation by reducing TLR4 and hydrogen peroxide levels [86].

**Fig. 11:**
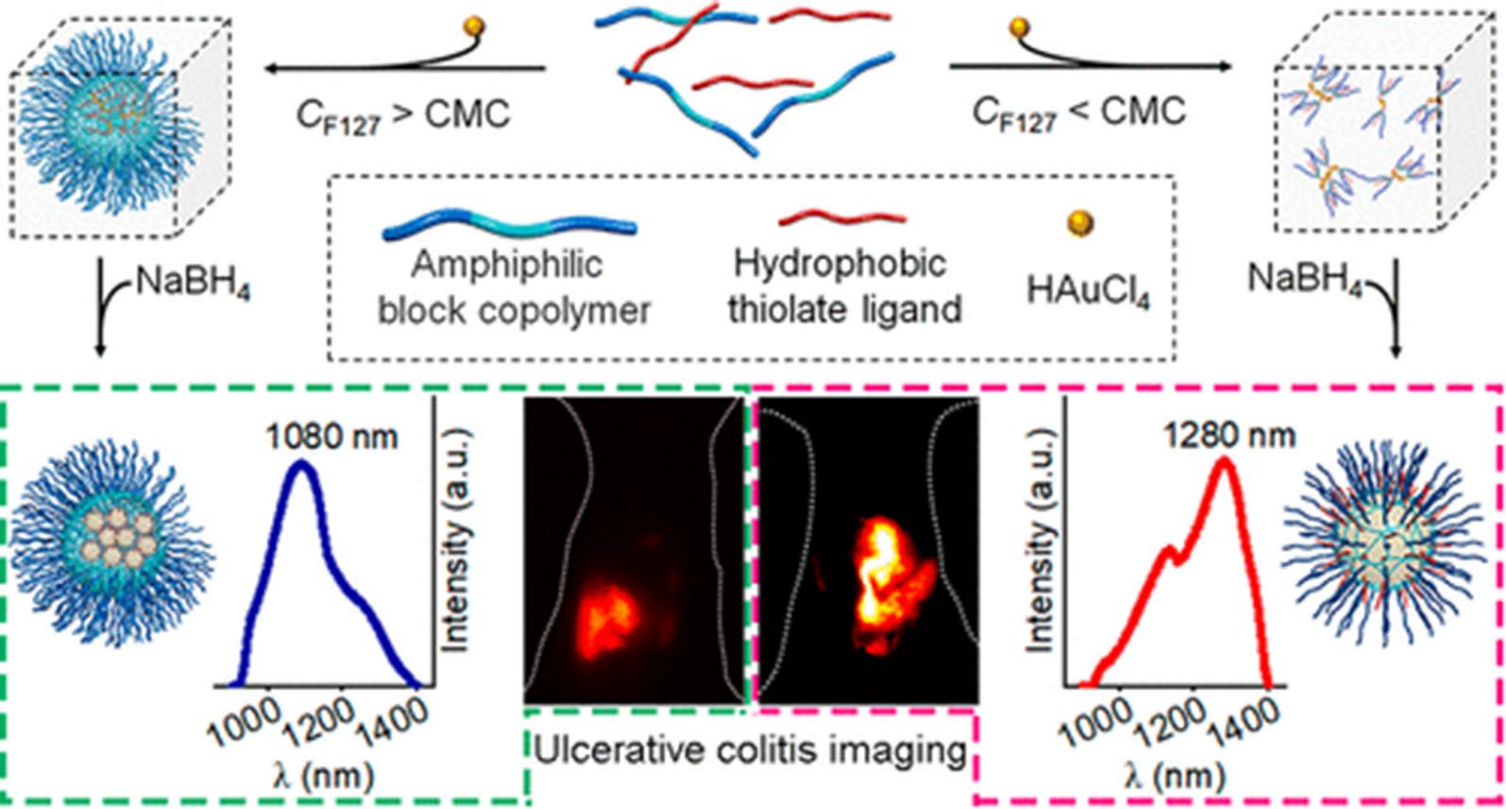
Schematic diagram of the synthesis process and mechanism of IR-II AuNPs (cited in Liu et al. [91])

The therapeutic effects of two different doses of AuNPs were evaluated by Rehab M Hussein and Hanan Saleh’s research team in an experimental colitis study involving rats. The findings demonstrated that AuNPs had a positive impact on the regenerative capacity of damaged tissues in the colon, suppressed inflammatory cytokine responses, and reduced colonic malondialdehyde levels and myeloperoxidase activity in dinitrobenzenesulfonic acid-induced colitis model rats. Furthermore, there was a significant enhancement observed in the antioxidant defence system [85]. In addition to their therapeutic effectiveness, AuNPs have also been utilized for imaging IBD. A research team led by Liu Jinbin developed a method to enhance the emission and biointeractions of redshifted luminescent AuNPs using an amphiphilic block copolymer (ABC) template that allows for controlled hydrophobic interactions with various forms of monomers and micelles. Within the hydrophobic core of ABC micelles, uniform clusters of near-infrared II (NIR-II) AuNPs are formed in situ, exhibiting strong interparticle hydrophobic interactions and emitting light at 1080 nm with a high quantum yield (QY) of 1.6%. Conversely, rigid NIR-II AuNPs serve as the surface ABC monomer, leading to intense intraparticle hydrophobic interactions and resulting in redshifted emission at 1280 nm. This method enhances the affinity of these peptides for damaged colonic mucosa during colitis imaging (Fig. 11) [87].

#### Other metal materials

**Fig. 12:**
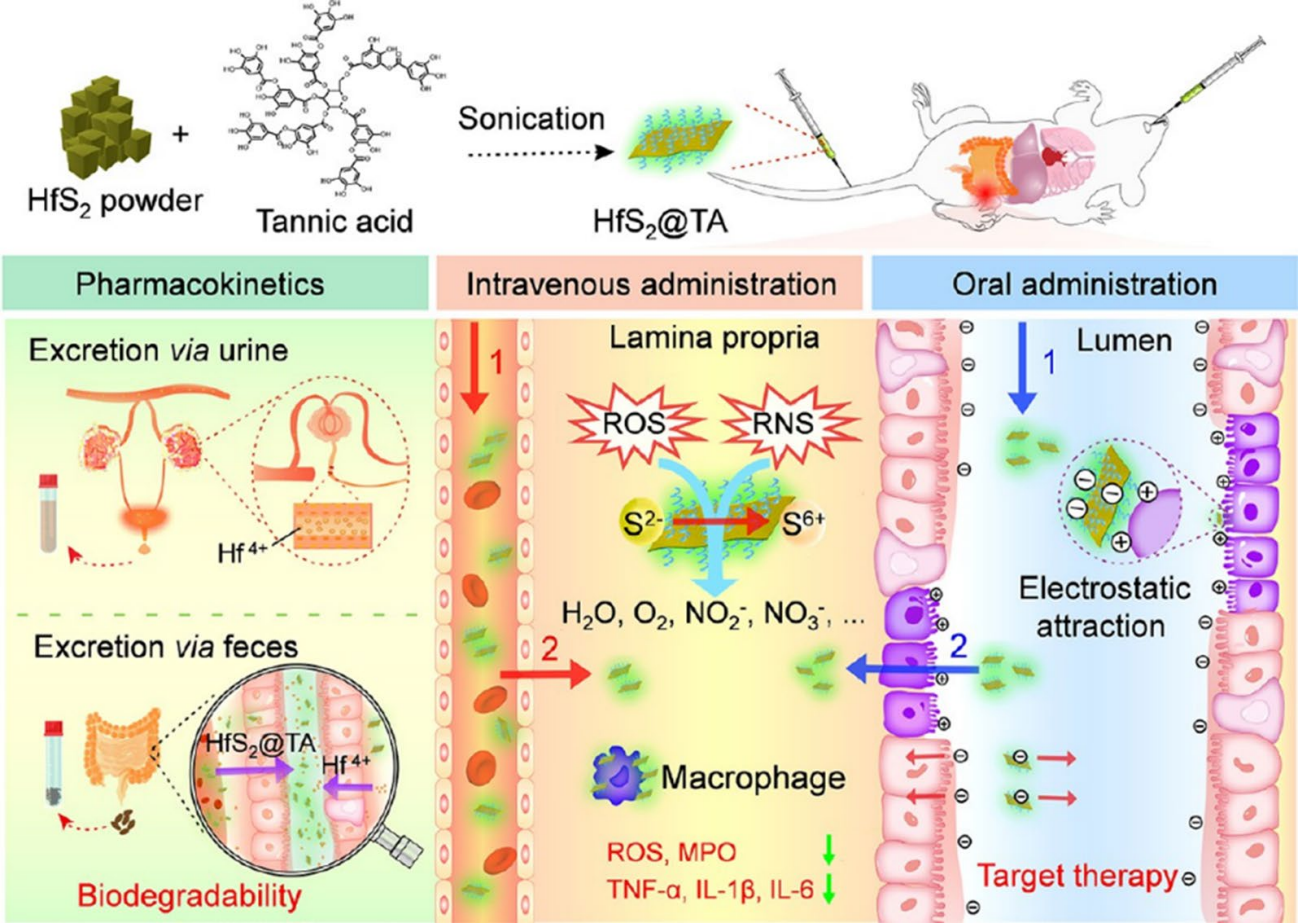
Schematic illustration of biodegradable HfS_2_@TA atomic crystal stripping for targeted IBD therapy (cited in Li et al. [98])

The utilization of nanomaterials in the management of IBD can be traced back to the development of Prussian blue NPs (MPBZs). MPBZs are synthesized by mixing a Mn^2+^ solution with polyvinylpyrrolidone (PVP) and [Fe (CN)_6_]^4−^ solution. Given that Mn(II) and Fe(II) ions possess reductive properties, they can eliminate ROS and mitigate intestinal damage [88]. Molybdenum-based nanomaterials, such as molybdenum disulfide and molybdenum phosphates, have garnered considerable attention due to their excellent biocompatibility and effective antioxidant capabilities [89, 90]. Chen Zhang and colleagues developed a novel oral zero-valent molybdenum nanoparticle (ZVMNs) for treating IBD. Both in vitro and in vivo experiments confirmed the therapeutic effects of ZVMNs in a murine model of IBD. These NPs, synthesized via a sonication method, are approximately 3 nm in size. RNA sequencing of mouse colonic tissues exposed to molybdenum NPs revealed downregulation of intestinal inflammatory genes and inhibition of the NF-κB signalling pathway, which plays a role in inflammation [91]. Molybdenum, a transition metal, possesses distinct physical and chemical characteristics that have been extensively investigated in various fields, including chemistry, functional electronics, mechanics, physics, energy materials, and biomedicine. These nanomaterials in the form of thin-sheet flakes exhibit the ability to react with ROS even under low light conditions. In a murine model of IBD, polyethylene glycol-modified Mo_3_Se_4_ nanoflakes (PMNFs) not only cleared ROS and inhibited the NF-κB signalling pathway but also downregulated tight junction proteins (ZO-1, ccluding, and claudin-1), reduced mucin-2 (MUC2) levels, and induced apoptosis in epithelial cells caused by DSS [92]. The research group led by Liang Cheng employed a two-step delamination strategy using hydrofluoric acid (HF), tetrapropylammonium hydroxide (TPAOH), and titanium aluminium carbide (Ti_3_AlC_2_) powder to synthesize two-dimensional Ti_3_C_2_ nanosheets (NSs). These NSs remained stable under simulated gastric fluid (SGF) conditions, exhibiting excellent biocompatibility and the ability to clear various ROS. This straightforward synthesis method expands the application of NSs in colitis treatment [93]. Additionally, there is a growing trend of using nanomaterials developed for intestinal inflammation through electrostatic attraction. For instance, the clinical application of hafnium-based NPs has gained attention. Zha Zhengbao’s research group employed tannic acid-coated hafnium disulfide (HfS_2_@TA) nanoflakes as materials and employed a liquid-phase exfoliation method to prepare two-dimensional atomic crystals for targeted therapy of IBD. By leveraging the interconversion of the S^2−^/S^6+^ oxidation states, the HfS_2_@TA nanoflakes obtained exhibit dual functionality in effectively eliminating ROS/RNS and reducing the production of inflammatory factors. Encouragingly, following oral or intravenous administration, HfS_2_@TA rapidly suppressed inflammation and restored the intestinal mucosal barrier in an IBD animal model. These findings highlight the potential of ultrathin atomic crystals such as HfS_2_@TA in the colon for targeted therapy against IBD (Fig. 12) [94].

### Natural products

**Fig. 13:**
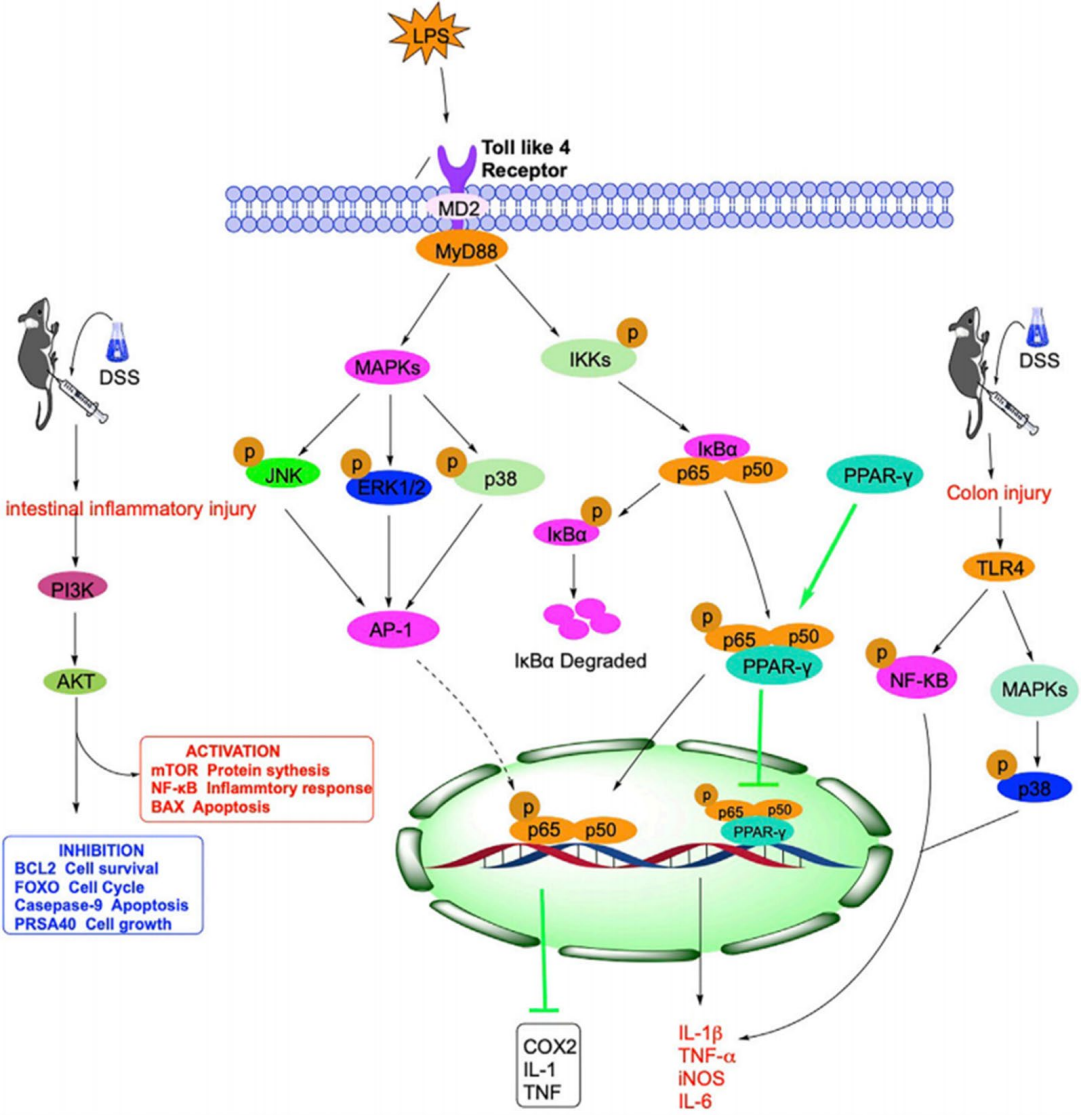
The mechanism of the signaling pathway related to IBD, such as NF-CAMb, TLR4, PPAR, PI3K, etc. (cited in Yuan et al. [100])

Natural products refer to a variety of biologically active extracts or isolated metabolites derived from traditional Chinese medicine. They have gained considerable attention due to their unique advantages, such as minimal adverse reactions, stable therapeutic effects, flexibility in dose adjustment, broad applicability, and a wide range of target actions [95]. Current research suggests that natural products may treat IBD mainly by operating through various signalling pathways, such as the NF-кB, TLR4, PPAR, and PI3K signalling pathways. They can delay oxidative stress, inhibit NLRP3 inflammasome activation, preserve intestinal barrier function, and regulate the gut microbiota, as depicted in Fig. 13 [96].

However, natural medicines face limitations in terms of solubility, permeability, and bioavailability, which restrict their use when administered using traditional methods. The micro- and nanoformulations of these natural medicines, such as those linked with metal ions, can improve their physical and chemical properties. This approach represents a new strategy to overcome these limitations. The following sections provide an overview of recent advancements in the use of natural products for IBD treatment and explore how nanotechnology can offer novel insights and directions for harnessing the potential of natural products in preventing and treating IBD.

#### Procyanidins

Procyanidins, known as natural polyphenolic compounds, can be found in the bark, seeds, cores, flowers, and leaves of various plants. Among these sources, peanut skin procyanidins (PSPCs) are particularly noteworthy due to their unique functional properties. Unlike grape seed procyanidins, which mainly consist of B-type dimers, PSPCs primarily exist as A-type dimers. Notably, PSPCs have a higher bioavailability than grape seed procyanidins. These polyphenols derived from peanut skins offer numerous health benefits through their antioxidative, antimicrobial, and anticancer properties. A study conducted by Wang Na’s team aimed to investigate the anti-inflammatory effects of peanut skin procyanidin extract (PSPE) and peanut skin procyanidins (PSPC) using a mouse model of DSS-induced colitis. The findings demonstrated that both PSPE and PSPC supplementation reversed the weight loss induced by DSS treatment while reducing the DAI. Additionally, they increased the number of goblet cells and the expression level of claudin-1 in the colon. Moreover, treatment with PSPE and PSPC effectively suppressed inflammation and oxidative stress in the colon by downregulating IL-1β, TNF-α, and MDA. Furthermore, the administration of both PSPE or PSPC significantly altered the gut microbiota composition, leading to increased production of short-chain fatty acids (SCFAs) in mice with colitis [97].

Silk fibroin protein (SS) has gained considerable attention as a drug nanocarrier due to its excellent biocompatibility and bioactivity. A team led by Wang utilized a one-step method to load procyanidins (PAC) into SSs, forming an SS/PAC composite material. SS/PAC exhibited uniform dispersion in aqueous solutions, high antioxidant capacity, and excellent biocompatibility. Through the modulation of oxidative stress, suppression of inflammatory responses, and reversal of tissue pathological damage, SS/PAC effectively alleviated the symptoms of DDS-induced ulcerative colitis [98].

**Fig. 14:**
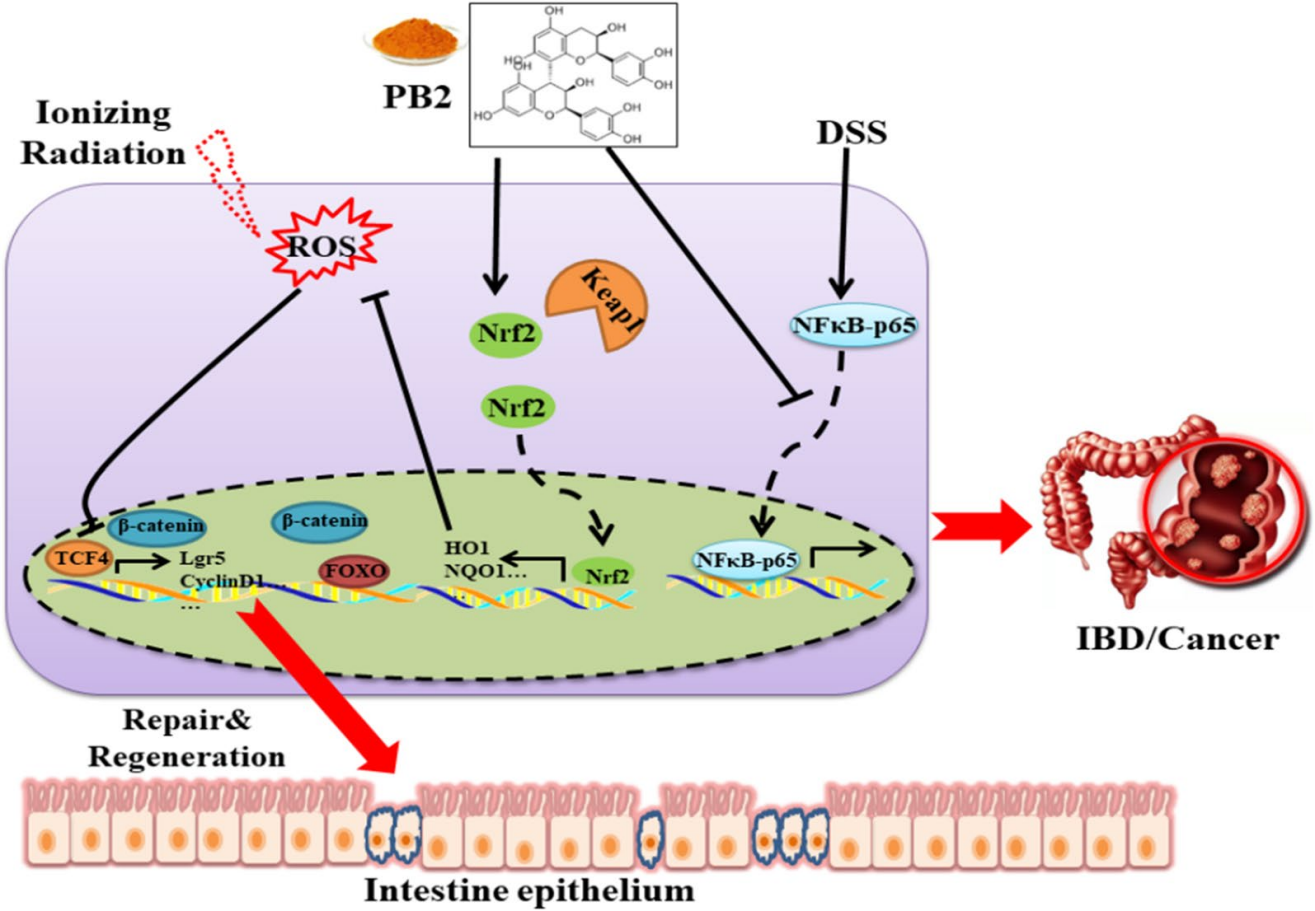
The schematic diagram summarizes the action mechanism of proanthocyanidin B2. In mice exposed to radiation, the level of oxidative stress in intestinal stem cells accumulated excessively, which led to the inactivation of Wnt signal and intestinal epithelial injury. PB2 increases the expression of antioxidant enzymes and decreases ROS levels by activating Nrf2. In addition, PB2 promotes intestinal stem cell activity by improving Wnt activation, which in turn promotes epithelial cell regeneration. In a DSS-induced colitis model, PB2 also promotes intestinal epithelial repair and reduces inflammation by reducing the nuclear localization of p65. Finally, PB2 treatment in a DSS induced colitis model chronically slowed the development of colitis-associated tumors (cited in Zhu et al. [104])

Procyanidin B2 (PB2), a member of the procyanidin family, is known for its potent antioxidant properties. PB2 is relatively stable under conditions of gastric and duodenal digestion and can protect human colon cells from oxidative damage in vitro. Xiangzhan Zhu and colleagues conducted experiments using a radiation-induced intestinal injury model and a DSS-induced colitis model. They found that PB2 could reduce the accumulation of ROS, protecting the intestines from radiation damage. PB2 effectively slowed the degradation of Nrf2 and significantly promoted Nrf2 translocation into the cell nucleus, which consequently upregulated the expression of antioxidant enzymes. Moreover, PB2, through enhanced Wnt/β-catenin signalling, facilitated regeneration driven by Lgr5-positive intestinal stem cells (ISCs), which was dependent on Nrf2 signalling activation. Additionally, PB2 alleviated experimental colitis and colitis-associated cancer by inhibiting the nuclear localization of p65 in a long-term inflammation model (Fig. 14) [99].

#### Curcumin

Curcumin, derived from the rhizomes of turmeric, is a polyphenolic compound with hydrophobic properties. It has diverse biological and pharmacological properties, such as anti-inflammatory, antioxidant, and anticancer properties [100, 101]. This compound has been extensively researched in the context of IBD and related diseases. Curcumin exerts its effects through multiple mechanisms, such as regulating M1/M2 macrophage polarization, inhibiting TLR signalling pathway interference with macrophage polarization and effector functions, inhibiting NLRP3 inflammasome activation, modulating the balance of Treg/Th17 cells, and downregulating members of the NF-κB signalling pathway, thereby inhibiting the release of inflammatory cytokines and reducing oxidative stress levels [102, 103].

Research conducted by Akira Andoh’s group has shown that the progression of colitis in mice can be effectively inhibited by curcumin NPs. The administration of curcumin NPs resulted in a significant reduction in the DIA and mitigating weight loss. Moreover, treatment with curcumin NPs led to a notable suppression of nuclear factor κB (NF-κB) production in colonic epithelial cells. Additionally, an increase in the accumulation of bacteria responsible for producing butyrate was observed after treatment with curcumin NPs [104]. Bo Xiao’s research team successfully integrated pH-responsive Eudragit S100 (ERS100)/PLGA microparticles (MPs) to achieve the sustained and specific release of curcumin in the colon. The study involved synthesizing microparticles with sizes ranging from 1.5 to 1.9 μm and loading them with a minimal amount of PLGA. This innovative system demonstrates promising potential for precisely delivering curcumin to damaged colon tissues in a controlled and efficient manner [105].

In a separate study conducted by Ana Beloqui and her team, the authors assessed the effectiveness of combining pH-sensitive PLGA with methylmethacrylate sulfonate polymer (ERS 100) that contained curcumin NPs. Researchers have ensured the targeted delivery of curcumin to the inflamed mucosa by encapsulating it within pH-sensitive NPs made from polymers. These curcumin NPs possessed favourable physicochemical properties, such as appropriate size and surface charge, which are crucial for delivering drugs to the colon. Moreover, these curcumin NPs successfully traversed the epithelial barrier of Caco-2 cells. Upon administration of these curcumin NPs, there was a significant decrease in TNF-α secretion in LPS-activated macrophages. Additionally, an in vivo experiment involving mice with DSS-induced colitis demonstrated a noteworthy reduction in MPO activity and TNF-α secretion after 8 days of treatment with curcumin NPs. This study highlights the promising application of curcumin for treating IBD [106].

#### Gallic acid (GA)

GA is a compound with multiple hydroxyl groups and a phenolic structure that has been found to possess anti-inflammatory, antioxidant, and antiviral properties [107]. GA can effectively downregulate the expression of P-IκBα and P-NFκBα while increasing the levels of IL-4 and IL-10. Additionally, it downregulates the expression of various cytokines, such as IL-6, IL-12, IL-17, IL-23, TGF-β, and TNF-α, in ulcerative colitis, thereby reducing inflammation [108]. Furthermore, GA acts as an effective antioxidant by inhibiting p65-NF-kB nuclear translocation, pSTAT3Y705 activation, and IκB degradation in the colonic mucosa. Additionally, it reduces MPO, iNOS, and COX2 levels in colon tissues, resulting in improved intestinal epithelial damage [109].

#### Astaxanthin (AX)

AX is a fat-soluble pigment known for its excellent antioxidant, anti-inflammatory, and immunomodulatory properties [110]. However, there are several challenges regarding the oral administration of AX, such as poor water solubility and gastrointestinal instability, which limit its effectiveness in intervening in IBD [111]. Encapsulating AXs in NPs can protect them from the effects of the gastrointestinal environment and allow them to accumulate in inflamed colonic tissues [112]. Tian Mingqian and colleagues initially employed a combination of whey protein isolate (WPI) and dextran (DX) to create AX NPs. These NPs were further modified with (3-carboxypentyl) (triphenyl) phosphonium bromide (TPP). To enhance their effectiveness, the surface of the NPs was coated with HA. In vivo experiments demonstrated that these targeted AX NPs effectively reduced inflammation by modulating the TLR4/MyD88/NF-κB signalling pathway. Additionally, they positively influenced the composition of the gut microbiota and promoted the production of SCFAs. The precise delivery of AX to mitochondria was achieved through the strong affinity of HA for the CD44 receptor [119].

**Fig. 15:**
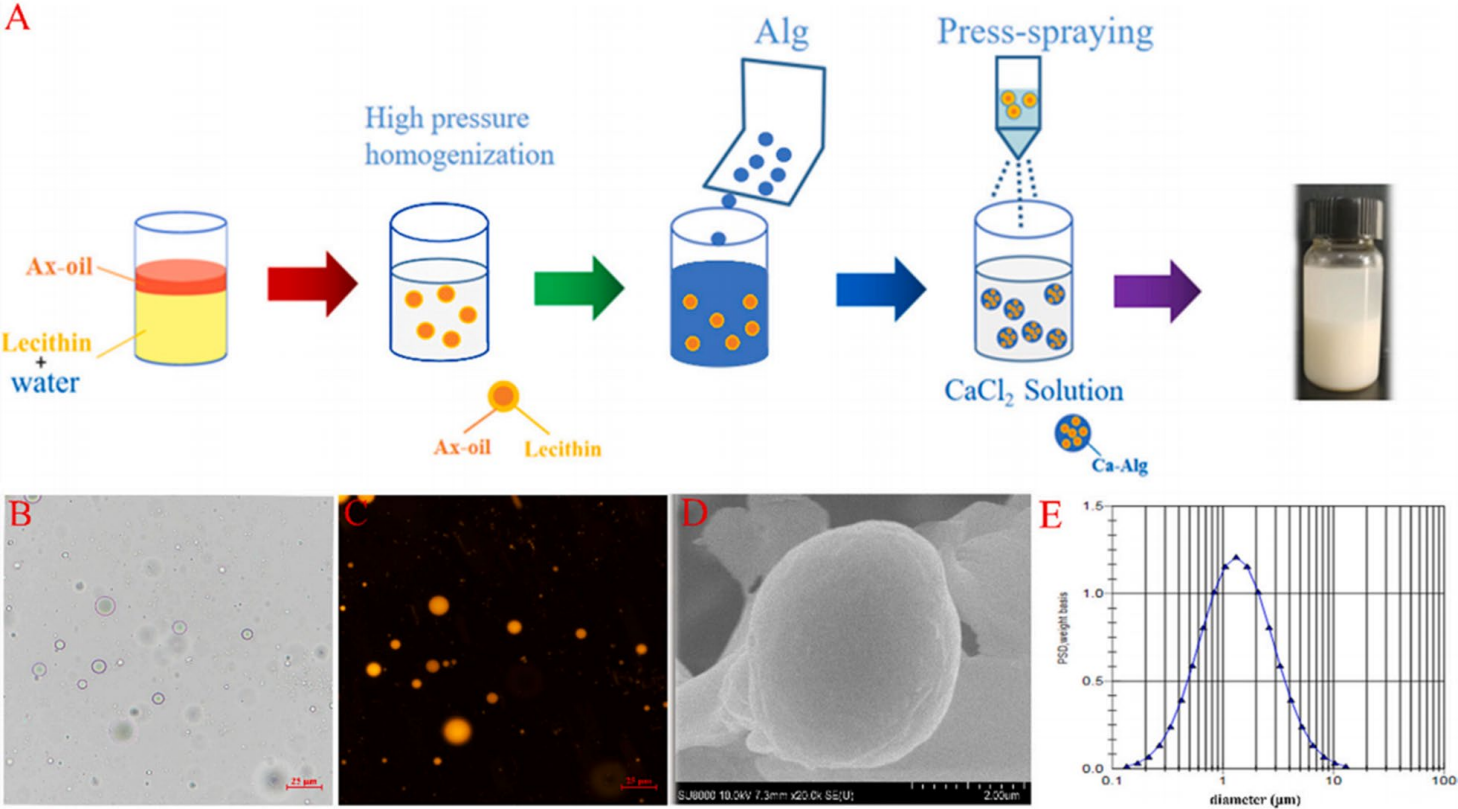
Preparation and characterization of Ax-Alg. **A** Representative images of Ax-Alg in optical and fluorescence microscopy (**B**, **C**) and cryo-scanning electron microscopy (**D**). Size distribution of Ax-Alg (**E**) (cited in Zhang et al. [120])

Ai Chunqing’s research group used high-pressure spraying and ion gelation to prepare colon-targeted alginate particles rich in AX (Ax-Alg). Ax-Alg could tolerate oral, gastric, and small intestinal environments and reach the colon for AX release through fermentation by the gut microbiota. Experiments on mice have shown that Ax-Alg can regulate the gut microbiota composition by decreasing the abundance of Enterobacteriaceae family members, which are known to be associated with the development of ulcerative colitis. The therapeutic effect of Ax-Alg was found to be more effective than oil-in-water emulsion due to the combined action of AX and alginate. Additionally, Ax-Alg has been shown to increase IL-10 levels while reducing IL-6 and IL-1β levels (Fig. 15) [113].

#### Luteolin

Luteolin is a natural flavonoid with excellent anti-inflammatory and free radical scavenging activities [114]. Luteolin significantly reduces MDA levels, enhances antioxidant capacity by activating the Nrf2 signalling pathway, and inhibits the expression of P-STAT1 and P-JAK1, thereby blocking the NF-κB pathway. Luteolin can upregulate the expression of OCLN, CLDN1, and ZO1 while decreasing the levels of P-STAT3 and CLDN2 by blocking the STAT3 signalling pathway. Luteolin significantly alleviates the symptoms of DSS-induced ulcerative colitis in mice. In vitro cell models have demonstrated that it can upregulate SHP-1, reduce STAT3 phosphorylation and significantly enhance intestinal mucosal barrier function [115].

Chen Tan’s research team developed NPs that respond to ROS and precisely deliver luteolin to specific locations affected by colitis. The NPs aided in removing ROS and inhibiting the release of proinflammatory cytokines such as IL-17A, IL-6, interferon-γ, and tumour necrosis factor-α. They also enhanced glutathione levels and promoted the production of anti-inflammatory cytokines such as IL-10 and IL-4. Additionally, these NPs can regulate the balance of helper T cells, thereby controlling the inflammatory environment [116].

#### Quercetin

Quercetin is a flavonoid compound extracted from plants [117]. It possesses anti-inflammatory and antioxidant activities and exerts therapeutic effects on intestinal inflammation when orally administered [118, 119].

A team led by Castangia developed vesicles coated with chitosan/nutriosomes and loaded with quercetin. The in vitro release profiles of quercetin at various pH levels demonstrated a notable enhancement in its accumulation specifically within the colon, enabling localized antioxidant and anti-inflammatory effects at the lesion site. This delivery system reduced colonic damage and significantly ameliorated TNBS-induced colitis [120].

Silk fibroin NPs have been used as reversible carriers for biologically active macromolecules and small polyphenolic compounds such as resveratrol or curcumin in nanomedicine [121–123]. Patricia and colleagues prepared silk fibroin NPs loaded with quercetin. Quercetin effectively decreased the levels of proinflammatory cytokines (TNF-α, IL-1β, and IL-6), the chemokine MCP-1, and the cellular adhesion molecule ICAM-1. Moreover, it significantly downregulated the expression of NLRP3 in the colon only when it was loaded into NPs [124].

#### Baicalein

*Scutellaria baicalensis* contains more than 40 known types of flavonoid glycosides and aglycones. The major constituents include baicalin, baicalein, wogonoside, and wogonin, with baicalin being the active component extracted from the dry roots of the plant [125].

The inhibitory effects of baicalin on the polarization of M1 macrophages induced by lipopolysaccharide have been observed, while baicalin also promotes the transition toward the anti-inflammatory M2 macrophage phenotype. Additionally, an increase in interferon regulatory factor 4 (IRF4) expression leads to the differentiation of macrophages into anti-inflammatory M2-type macrophages, thereby alleviating symptoms associated with colitis^.^[126–128]. Baicalin effectively inhibits the release of IL-6, PEG2, IL-1β, and TNF-α by obstructing the NF-κB and PI3K/Akt signalling pathways while simultaneously increasing IL-10 levels. Moreover, it significantly decreased the MDA, IL-1β, MPO, PEG2, and TNF-α levels in the colon tissues of rats with ulcerative colitis [129]. Baicalin exhibits the ability to downregulate the expression levels Caspase-3, Caspase-9, Bax, and FasL (Fas ligand) while simultaneously upregulating Bcl-2 expression, effectively hindering apoptosis [130]. To improve the function of the intestinal mucosal barrier, baicalin has been found to address the disrupted composition of gut microbiota caused by TNBS. This is achieved by reducing the ratio of Firmicutes-to-Bacteroidetes, increasing levels of butyric acid, and regulating the metabolism of short-chain fatty acids [127].

Notably, the combined utilization of baicalein and baicalin demonstrates enhanced outcomes in comparison to their individual usage, with baicalin showcasing a more potent safeguarding impact on the gastrointestinal tract [131]. These research results indicate that *Scutellaria baicalensis* has the potential to be a beneficial treatment option for IBD. Nevertheless, investigations have demonstrated that administering high doses of baicalein to female mice results in notable foetal chromosomal abnormalities and a substantial rise in maternal weight [132]. Therefore, the application scope and limitations of *Scutellaria baicalensis* in IBD patients require further investigation.

#### Silymarin

SM, also known as silybin, is a flavonoid compound extracted from the milk thistle plant that has anti-inflammatory properties. However, its bioavailability is limited due to its rapid degradation, poor intestinal epithelial cell permeability, and low water solubility. Thu-Ha Thi Nguyen and colleagues developed silicon dioxide redox NPs (siRNPs) with diameters ranging from 50 to 60 nm to enhance the uptake and delivery of SM in the bloodstream, thereby improving its bioavailability in colonic tissues. The antioxidant capacity and anti-inflammatory effectiveness of SM@siRNP were significantly enhanced in vitro through the inhibition of NO and proinflammatory cytokine production. Oral administration of SM@siRNP greatly improved the absorption and retention of SM in the colon mucosa. Additionally, treatment with SM@siRNP effectively alleviated colonic mucosal damage in mice with DSS-induced colitis [133].

#### Kaempferol

**Fig. 16:**
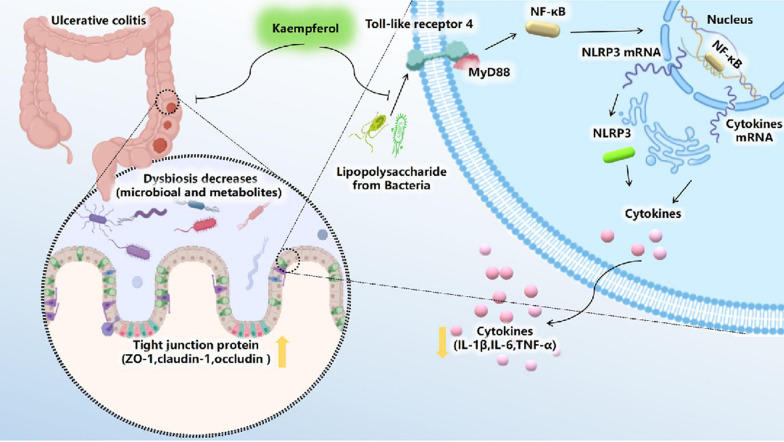
KAE exerts excellent anti-UC effects through the gut microbial pathway associated with the LPS-TLR4-NF-κB core pathway. By inhibiting the proliferation of pathogenic gram-negative bacteria, KAE changes the metabolic profile, blocks the activation of the NF-kB pathway, improves the integrity of intestinal tight connections, inhibits inflammatory factors, and increases antioxidants, thereby reducing colon inflammation caused by DSS (cited in Qu et al. [141])

Kaempferol, a flavonoid compound found in medicinal plants, has been shown to possess anti-inflammatory and antioxidant properties. In a study conducted by Qu Yifan and colleagues, the effects of kaempferol on DSS-induced colitis in C57BL/6 mice were investigated. The results showed that kaempferol was able to prevent disruption of the intestinal barrier caused by DSS through increasing the levels of ZO-1, Occludin, and Claudin-1. Additionally, it reduced proinflammatory cytokines such as IL-6, IL-1β, and TNF-α while enhancing the expression of the IL-10 mRNA. Furthermore, kaempferol was observed to reshape the gut microbiota by increasing the Firmicutes/Bacteroidetes ratio and decreasing Proteobacteria abundance after DSS treatment in mice. Overall, these findings suggest that kaempferol may exert its protective effects on colitis mice via modulation of the gut microbiota and TLR4-NF-kB signalling pathway (Fig. 16) [134].

#### Anthocyania

Anthocyanins, which are plant pigments that can dissolve in water, can be found in different parts of higher plants. These pigments possess antioxidant activity, antibacterial effects, and the ability to reduce inflammation. In the treatment of inflammatory bowel disease (IBD), anthocyanins have a positive impact by safeguarding the integrity of the intestinal mucosa, restoring the function of the protective barrier formed by epithelial cells, regulating the composition of the gut microbiota, and exerting anti-inflammatory effects [135].

Previous research has indicated that the intake of foods rich in anthocyanins significantly enhances the production of mucins and proteins involved in wound healing, such as MUC1, MUC2, MUC3, CDC42, RAC1, GAL2, GAL3, GAL4, and Relmβ. These proteins play vital roles in facilitating the repair processes of mucosal injuries [136, 137]. Tight junctions (TJs) play a crucial role in regulating the movement of molecules between epithelial cells by forming a barrier. Different TJ proteins have specific functions, with Claudin1 and Claudin4 enhancing the tightness of epithelial cells. On the other hand, Claudin2 may contribute partially to the formation of intermittent TJ strands, potentially impacting the absorption of large antigenic molecules in the lumen [138–140]. Occludin, which plays a role in cell adhesion, is responsible for regulating the permeability of the paracellular pathway. ZO-1, a well-known marker of tight junctions (TJs), functions as a crucial link between occludin and claudin and the actin cytoskeleton to enhance the integrity of the epithelial barrier [141, 142]. Yujia Peng and colleagues have made the discovery that goji berry-derived anthocyanins possess the ability to enhance the levels of tight junction proteins (ZO-1, Occludin, and Claudin-1) and their corresponding mRNAs. These anthocyanins exhibit significant inhibitory effects on pro-inflammatory cytokines such as TNF-α, IL-6, IL-1β, IFN-γ, MCP-1, LPS, and PGE2, as well as their respective mRNAs [143].

**Fig. 17:**
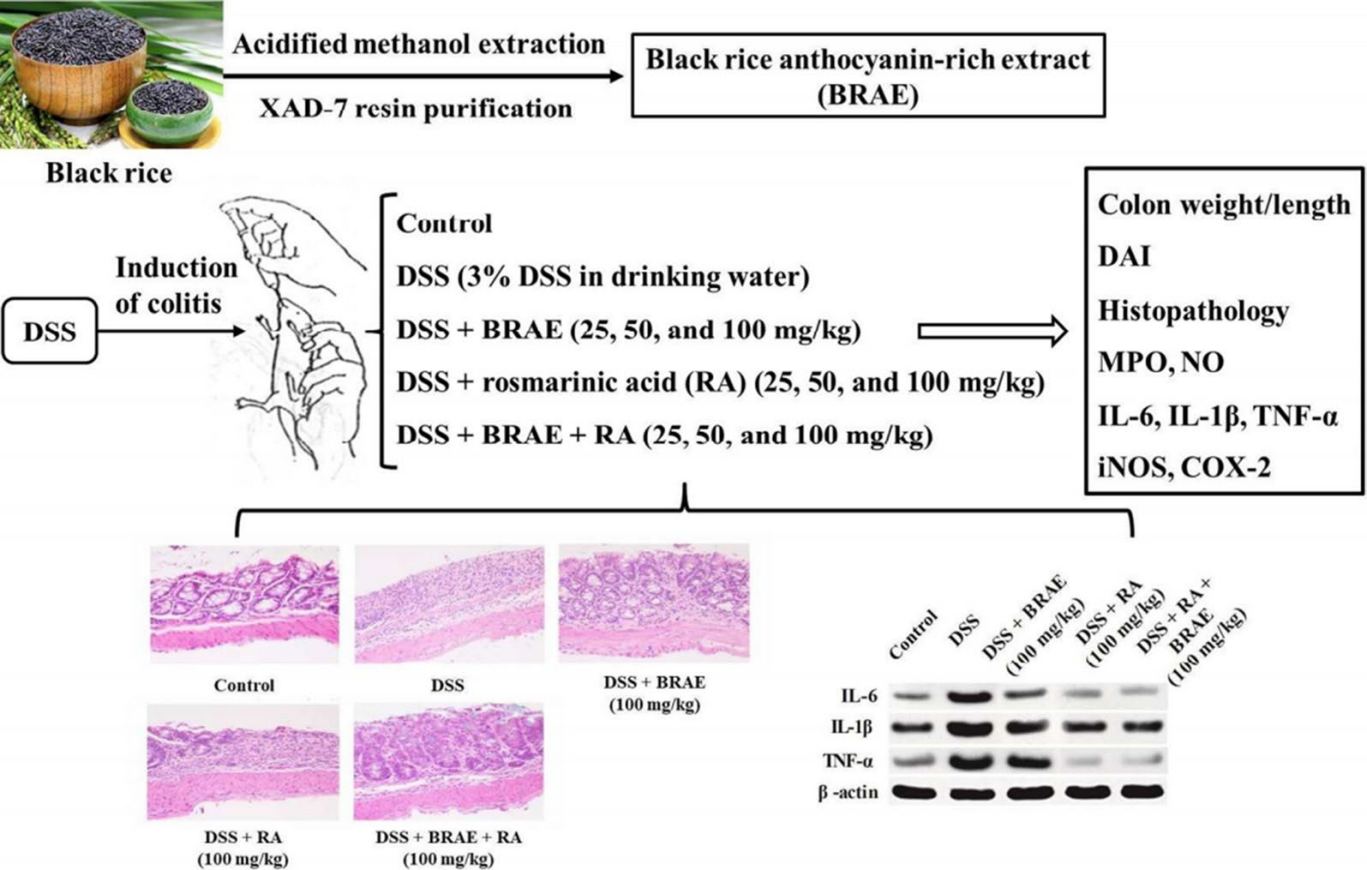
Black rice anthocyanins extract and rosemary acid IBD. The anti-inflammatory mechanism in the treatment of black rice anthocyanins extract and rosemary acid by cutting inflammatory mediators to improve DSS induced colitis in mice (cited in Zhao et al. [152])

Classical drugs such as 5-ASA and blueberries can suppress the expression of colonic COX-2. However, anthocyanins more prominently regulate iNOS, reduce leukocyte infiltration, and enhance colonic antioxidant defense capabilities [144]. Extracts from black rice and purple yam tubers can downregulate the expression of TNF-α and myeloperoxidase (MPO) (Fig. 17) [145, 146]. These findings indicate that anthocyanins possess anti-inflammatory properties through the regulation of cytokine transcription and translation, suppression of pro-inflammatory cytokines, and enhancement of anti-inflammatory cytokines.

Moreover, anthocyanins extracted from blackcurrant fruit promote the growth of Bifidobacterium members in the intestinal microbiota, reduce the ratio of Firmicutes to Bacteroidetes, and increase the production of SCFAs, thereby exerting a regulatory effect on the gut microbiota [143]. 16S rRNA amplicon sequence analysis showed that mulberry anthocyanin (MAS) mitigated the gut microbiota dysbiosis induced by DSS, manifested by a reduction in *Escherichia coli* abundance and an increase in the abundance of Akkermansia, Muribaculaceae, and Allobaculum members [147].

#### Resveratrol

**Fig. 18:**
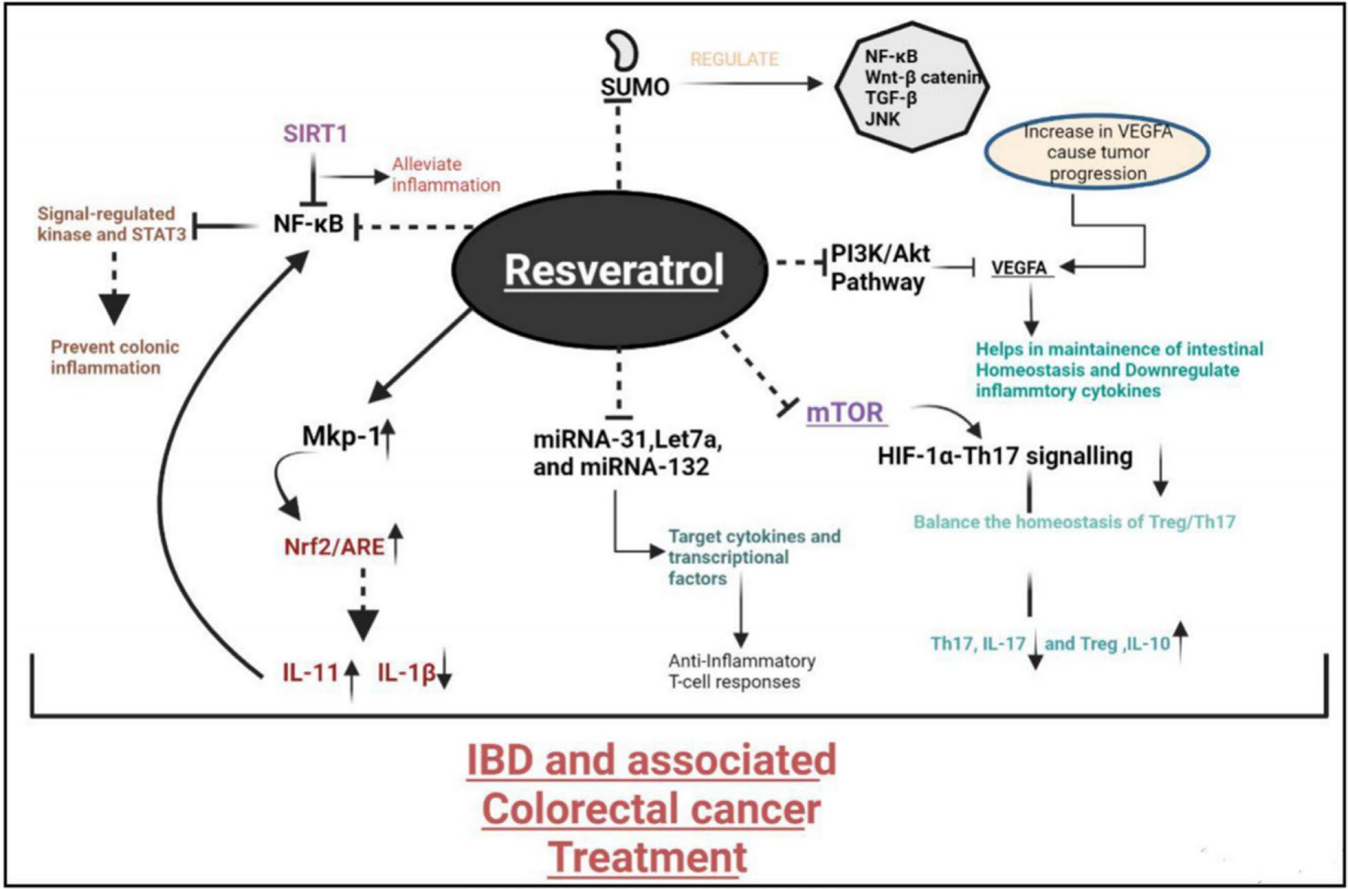
Anti-inflammatory mechanism of resveratrol in IBD treatment. *ARE *antioxidant response element, *Akt* protein kinase B, *HIF-1α* hypoxia-inducing factor 1-α, *ILs* interleukin, *JNK* c-Jun N-terminal kinase, *mTOR* mammalian target of rapamycin, *NF-kB* nuclear factor-κB, *Nrf2* transcription factor NF-E2 related factor 2, *PI3K* phosphoinositol 3-kinase, *SIRT1* Sirtuin 1, *STAT3* signal sensor and transcriptional activator 3, *TGF-β* transforming growth factor β, *TREG* regulatory T cell, *VEGFA* vascular endothelial growth factor A. (Quoted in Gowd et al. [155])

Resveratrol, a natural polyphenol present in grapes and berries, has been extensively studied for its potential therapeutic effects on IBD in various animal models. Resveratrol exhibits beneficial properties by interacting with multiple molecular targets, such as NF-kB, SIRT1, mTOR, HIF-1α, miRNAs, and TNF-α. These interactions effectively mitigate or prevent symptoms associated with IBD (Fig. 18). However, the clinical application of resveratrol is hindered due to challenges related to its low solubility in water, chemical instability, limited bioavailability, and rapid degradation within the body [148].

#### Polysaccharides

The role of natural polysaccharides in ulcerative colitis has received increasing attention. Natural polysaccharides such as hawthorn, astragalus, and aloe vera have been shown to have excellent anti-inflammatory effects, intestinal barrier repair, and gut microbiota regulation properties and have been reported to effectively alleviate ulcerative colitis. Natural polysaccharides offer advantages such as high biocompatibility and biodegradability and excellent controlled release capabilities, making them common choices as nanocarriers. Moreover, polysaccharides cannot be digested in the gastrointestinal tract and can safely reach the colon as prebiotics, protecting drugs from digestion by gastrointestinal fluids [149].

#### Reed polysaccharides (PRP2s)

Liu Kehai’s research team, among others, isolated low-selenium (PRP2-SENPS-L) and high-selenium (PRP2-SENPS-H) PRP2s. Utilizing electrostatic attraction, they constructed a PRP2-based sulphur thiopurine liposome nanodrug delivery system (PRP2-SENPS-H/Aza lips). The PRP2 nanocarrier can protect sulphur thiopurine liposomes from digestion in the gastrointestinal tract, enhancing their therapeutic effect on ulcerative colitis while significantly reducing the liver damage caused by sulphur thiopurine. This delivery system improves the clinical efficacy of sulphur thiopurine liposomes [150].

#### Arctiin

Arctiin is a lignan compound belonging to the Asteraceae family, and it is a well-known medicinal plant that grows worldwide, especially in Southeast Asia. Arctiin has been used to treat inflammation, infections, metabolic disorders, and various central nervous system dysfunctions. This natural compound has been demonstrated to exert its anti-inflammatory effects by inhibiting NO production through the suppression of iNOS at both the protein expression and activity levels [151, 152]. Furthermore, the ERβ/TRIM21/PHB1 pathway is inhibited by arctiin, which helps to preserve the integrity of the mucosal barrier by preventing goblet cell apoptosis. Additionally, arctiin has been found to enhance colonic inflammation reduction through its ability to downregulate Th1 and Th17 cell differentiation via the mTORC1 pathway [153, 154].

#### Andrographolide sodium bisulphate (ASB)

ASB is a water-soluble sulfonic acid salt derived from arctigenin. The research conducted by Guan Fengkun’s team revealed that ASB effectively mitigates YAP-mediated inflammation in the colon and inhibits the release of proinflammatory factors by modulating the TLR4/MyD88/NF-κB pathway in the liver. These findings suggest that ASB exerts protective effects against DSS-induced ulcerative colitis (UC) and hepatic damage [155]. Andrographolide modulates the STAT3 signalling pathway, resulting in decreased levels of factors such as IL-23, IL-17, and IFN-γ in both serum and colonic tissues. Moreover, the reduction in the proportions of CD4+ cells, which are Th1 and Th17 clees, induced by ASB facilitates an anti-inflammatory response by augmenting Th2 activity [156]. In addition, the IL-4R/STAT6 signalling pathway is effectively inhibited by arctigenin lactone through its ability to prevent the specific binding between IL-4/IL-13 and IL-4R. This hindrance leads to a reduction in MPO activity and TNF-α secretion, thereby ameliorating symptoms caused by oxazolone (OXA)-induced ulcerative colitis [157].

#### Berberine

Berberine is a commonly utilized isoquinoline alkaloid for its anti-diarrhoeal properties. Previous studies have demonstrated that berberine has the ability to decrease the phosphorylation of STAT1 and STAT3, inhibit the NF-κB signalling pathway, suppress Th1/Th17 responses, and reduce the release of proinflammatory cytokines. Moreover, it promotes an increase in sIgA expression within the colon while maintaining immune response homeostasis [158].

Zhu Yubing’s research team successfully illustrated the activation of the PKB/SOCS1 signalling pathway by berberine. Additionally, they observed a decrease in p65 phosphorylation, leading to a reduction in the polarization of proinflammatory M1 macrophages. Furthermore, this compound has been found to effectively regulate the balance between M1 and M2 macrophages [159]. Li Chao’s research team developed nanomicelles containing carboxymethyl chitosan that encapsulated berberine (OC-B-BBR). OC-B-BBR significantly reduced colitis symptoms by modulating IL-6 expression and reshaping the gut microbiota. It also enhanced carbohydrate digestion and absorption, sugar metabolism, gluconeogenesis, and amino acid metabolism by increasing the abundance of beneficial bacteria and reducing the abundance of harmful bacteria. Furthermore, activation of the Nrf2 pathway and induction of P-glycoprotein expression are involved in the effectiveness of berberine in treating colitis [160–162].

#### Rhein

**Fig. 19:**
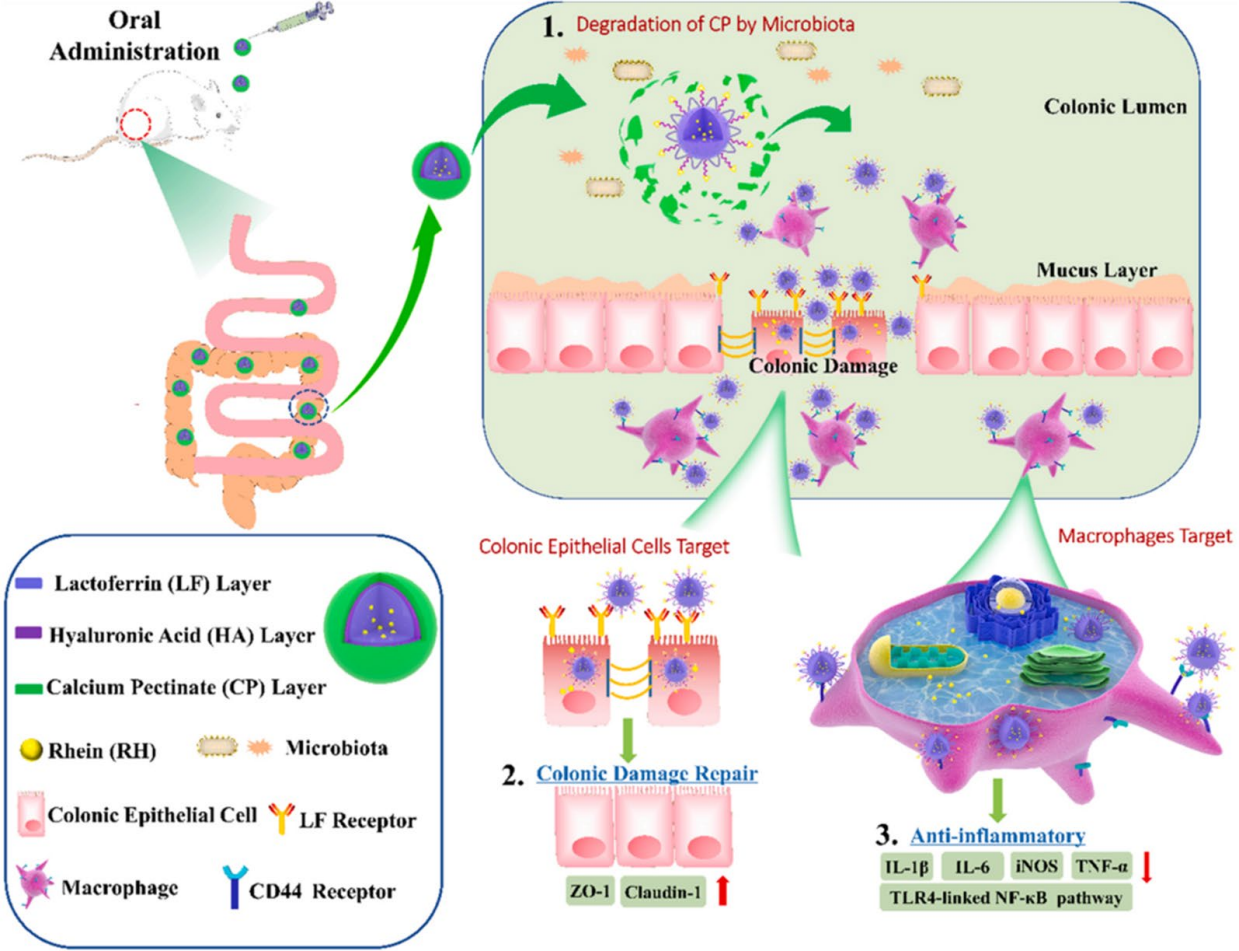
Schematic diagram of UC treatment in mice with CP/HA/RH-NPs. (1) Schematic diagram of oral CP/HA/RH-NPs via the GIT. CP can protect CP/HA/RH-NPs through the stomach and small intestine and further release HA/RH-NPs into the colonic lumen through degradation. (2) Schematic diagram of enhancing the effect of RH in repairing intestinal damage by targeting the expression of ZO-1 and Claudin-1 in UC mouse models through colonic epithelial cells. (3) The schematic of targeting macrophages can effectively promote the anti-inflammatory effect of RH through the TLR4/MyD88/NF-κB pathway and the anti-UC therapeutic effect in vivo. (Quoted in Luo et al. [160])

Luo Ruifeng and colleagues enhanced the targeting of rhein (RH)-loaded lactoferrin NPs using HA for electrostatic modification. The authors further improved the gastrointestinal stability of the carrier by coating it with pectin calcium (CP). These CP/HA/RH-NP NPs effectively alleviated ulcerative colitis and accelerated colonic healing by inhibiting the TLR4/MYD88/NF-κB signalling pathway (Fig. 19) [40, 41, 163].

## Conclusion and prospectives

IBD has emerged as a prevalent ailment that poses a substantial threat to human health. However, the pursuit of effective strategies to treat IBD remains a great medical challenge. Regrettably, within the confines of present clinical treatment modalities, we can only manage the symptoms of IBD, while the underlying root causes of the disease remain unaddressed. Consequently, in light of the aetiological considerations surrounding IBD and the burgeoning field of clinical nanomedicine, encompassing both inorganic NPs and natural product nanomedicine, this review explored their therapeutic potential in IBD.

First, despite the ongoing mystery surrounding the causes and development of IBD, compelling evidence suggests that there is a significant production of ROS and RNS within the inflamed colon. This excessive generation of ROS/RNS, along with subsequent oxidative stress and redox regulation disruption, plays a crucial role in driving the progression of IBD [25, 26]. This progression manifests as intestinal inflammation, mucosal damage, and the formation of mucosal ulcers. Consequently, the utilization of exogenous antioxidants to target excess ROS can restore redox balance, representing a promising therapeutic approach for mitigating IBD. Moreover, within the expansive landscape of China, numerous natural products have demonstrated the capacity to effectively treat IBD through diverse anti-inflammatory pathways. This direction aligns with the forefront of current nanomedicine development.

Moreover, inorganic NPs have broad application potential for treating IBD. These NPs exhibit remarkable catalytic performance, enzyme-like activity, and the capacity for varying metal chemical valences. Importantly, these compounds can directly engage with and neutralize ROS/RNS, thereby exerting a discernible therapeutic impact on oxidative stress-induced damage. Additionally, NPs are small in size and have a large surface area and a negative zeta potential, endowing them with excellent targeting capabilities within the intestinal environment, where inflammation leads to an increase in positively charged proteins [11–13]. Among these nanoparticles, selenium-loaded NPs are promising for colitis treatment due to their multifaceted antioxidant enzyme activity [53, 54]. Cerium oxide, which features variable oxidation states of cerium (Ce^3+^/Ce^4+^), plays a pivotal role in enhancing antioxidant activity [57–62]. Moreover, NP modifications include numerous polymer compounds designed to enhance biocompatibility and target specificity. Notably, tungsten-based NPs have emerged as promising therapeutics, exhibiting specific inhibitory effects on facultative anaerobic enterobacteria within the gut. This unique property contributes to the maintenance of intestinal barrier function and the regulation of the intestinal flora.

Owing to their distinctive structural characteristics, natural products actively participate in the modulation of inflammatory pathways. These pathways encompass crucial elements such as NF-κB, TLR4, PPAR, and PI3K, as well as other signalling cascades. Natural products contribute to a reduction in oxidative stress, inhibition of the NLRP3 inflammasome, preservation of intestinal barrier function, and regulation of the gut microbiome.

Both inorganic nanomaterials and natural products exhibit remarkable efficiency in clearing excess ROS/RNS, a factor contributing to the development of IBD. They exert unique effects on specific inflammatory pathways, suppress the inflammatory microenvironment, and rapidly restore intestinal barrier function. Looking ahead, inorganic NPs and natural products are expected to gain increasing popularity as novel therapeutic agents for the clinical treatment of IBD.

## Data Availability

Not applicable.

## Abbreviations

IBD: Inflammatory bowel disease
ROS: Reactive oxygen species
RNS: Reactive nitrogen species
EPR: Enhanced permeation and retention
GPx: Glutathione peroxidase
MSNs: Mesoporous silica nanoparticles
BNS: Biogenic selenium nanoparticles
SOD: Superoxide dismutase
CAT: Catalase
GSH: Glutathione
GST: Glutathione *S*-transferase
